# The antimicrobial peptide Angie 5 inhibits TcdA and TcdB from *Clostridioides difficile*

**DOI:** 10.1007/s00018-025-05799-2

**Published:** 2025-06-30

**Authors:** Stefanie Lietz, Lena-Marie Sokolowski, Katrin Lindner, Armando A. Rodríguez, Ludger Ständker, Verena Vogel, Barbara Spellerberg, Steffen Stenger, Daniel Alpízar-Pedraza, Katharina Ernst, Panagiotis Papatheodorou, Holger Barth

**Affiliations:** 1 Institute of Experimental and Clinical Pharmacology, Toxicology and Pharmacology of Natural Products, Ulm University Medical Center, 89081 Ulm, Germany; 2 Core Facility Functional Peptidomics, Ulm University Medical Center, 89081 Ulm, Germany; 3 Core Unit Mass Spectrometry and Proteomics, Ulm University Medical Center, 89081 Ulm, Germany; 4 Institute of Medical Microbiology and Hygiene, Ulm University Medical Center, 89081 Ulm, Germany; 5 Biochemistry and Molecular Biology Department, Center for Pharmaceutical Research and Development, Ave. 26 # 1605, Nuevo Vedado, Ciudad de La Habana, 10400 Cuba

**Keywords:** *Clostridioides difficile*, TcdA, TcdB, Toxin inhibitor, Endogenous proteins, Antimicrobial peptides, Angiogenin

## Abstract

*Clostridioides (C.) difficile* is a spore-forming, toxin-producing nosocomial human gut pathogen and a causative agent of gastrointestinal infections, leading to mild to severe diarrhea. Severe *C. difficile* infections (CDI) can cause life-threatening conditions, such as pseudomembranous colitis, colonic perforation, or toxic megacolon. The main virulence factors of *C. difficile* and responsible for CDI symptoms are two AB-type protein toxins, toxin A (TcdA) and toxin B (TcdB). TcdA and TcdB are large, single-chain proteins with multiple domains and glucosyltransferase activity. After receptor-mediated endocytosis, acidification of endosomes triggers insertion and pore formation of the toxins into the endosomal membrane for the delivery of their toxic glucosyltransferase domain (GTD) into the cytosol. There, the GTD glucosylates its target proteins, small GTPases of the Rho and/or Ras family, which leads amongst others to the collapse of the actin cytoskeleton and eventually to cell death. Here, we describe in silico predicted antimicrobial peptides, denoted as Angies, since they derive from the human endogenous protein angiogenin, as inhibitors for TcdA and TcdB. The strongest inhibitory capacity provided the derivative Angie 5, consistently in HeLa and Vero cells, as well as in the physiologically more relevant colon carcinoma cell line CaCo-2. Angie 5 delayed TcdA/TcdB-mediated glucosylation of its substrate proteins and, consequently, toxin-induced cell rounding as a consequence of actin-depolymerization. Moreover, the same Angie peptides that neutralized TcdA/TcdB also prevented the growth of *C. difficile *in vitro. In conclusion, our study paves the way for the development of antimicrobial peptide-based anti-toxin strategies to address *C. difficile*-associated diseases (CDADs).

**Supplementary Information:**

The online version contains supplementary material available at 10.1007/s00018-025-05799-2.

## Introduction

*Clostridioides (C.) difficile*, formerly known as *Clostridium difficile*, is a gram-positive, spore-forming, and clinically relevant (nosocomial) bacterial pathogen of the human gut. *C. difficile* is the causative agent of gastrointestinal infections in humans, leading to mild to severe diarrhea. Severe cases can also develop into life-threatening conditions, including pseudomembranous colitis, colonic perforation, or toxic megacolon. *C. difficile* infections (CDI) are a major contributor to the healthcare burden and high costs [31].

Typically, CDI is triggered by a disrupted gut microbiome, usually after antibiotic treatment, when *C. difficile* spores in the gut are transformed into their vegetative form and begin to overgrow. The bacterium secretes two highly potent AB-type protein toxins, which belong to the family of clostridial glucosylating toxins (CGTs). The two toxins, namely toxin A (TcdA) and toxin B (TcdB), are the main virulence factors and the causative agents of symptoms associated with CDI [3, 53]. However, hypervirulent *C. difficile* strains which are associated with increased severity of CDI additionally produce a third toxin, the binary *Clostridium difficile* transferase toxin (CDT) which has ADP-ribosyltransferase activity [16].

TcdA (310 kDa) and TcdB (270 kDa) are large, single-chain proteins with multiple domains and glucosyltransferase activity. They are further classified as ABCD-type toxins and possess at least four functional domains essential for uptake and action within host cells: the enzymatically active glucosyltransferase domain (GTD) at the N-terminus (A-activity), the cysteine protease domain (CPD) (C-cleavage), the delivery and receptor binding domain (D-delivery), and the CROPs (combined repetitive oligopeptides) domain (B-binding) [26, 40].

A two-receptor model has been postulated for TcdA and TcdB, where both toxins bind to their specific receptors via the CROP domain or the preceding second receptor-binding domain [17, 45]. After binding of TcdA and TcdB to its receptors on cell surfaces, receptor-mediated and clathrin (TcdB) and/or PACSIN2 (TcdA) endocytosis occurs [11, 18, 41]. Acidification of the endosomes by vesicular adenosine triphosphatases (V-ATPases) triggers membrane insertion and pore formation of the toxins [4, 39]. This in turn allows the GTD and the CPD to translocate through the pore into the cytosol [26]. Next, the cytosolic molecule inositol hexakisphosphate (InsP6) binds and activates the CPD for autocatalytic cleavage and release of the GTD into the cytosol [13, 19], where it glucosylates and thereby inactivates small GTPases of the Rho and/or Ras family, including Cdc42, RhoA, and Rac1. In this reaction, a glucose moiety from the co-substrate UDP-glucose is covalently attached (mono-O-glucosylation) by the toxins to their target proteins. GTPases of the Rho family control important cellular functions, such as the regulation of the actin cytoskeleton, and their inactivation by TcdA/TcdB leads to cytopathic cell rounding as a typical morphological feature of the intoxication of cultured cell monolayers by both toxins and eventually to cell death [2, 21, 30]. The inactivation of GTPases contributes to a disruption of the intestinal barrier, to intestinal damage and to other clinical symptoms that characterize the disease profile.

Current guideline-recommended treatment options for CDI are antibiotics, such as oral vancomycin or fidaxomicin as first-line drugs. The antibody bezlotoxumab and faecal microbiota transplantation are additional treatment options for patients with multiple recurring episodes of CDI [42].

In addition to antibiotic therapy, the inactivation of toxins with small compounds and antimicrobial peptides is a promising therapeutic approach against CDI. Recent examples from our laboratory have been the use of licensed drugs, such as amiodarone and ambroxol, or human defensins for inhibiting TcdA and TcdB (summarized in [5]). Due to their natural origin, treatment strategies using endogenous antimicrobial peptides might result in a better safety profile, higher serum stability, or better distribution within the human body. As such, exploiting the human proteome and peptidome for the identification of novel treatment options is advantageous and promising.

Previously, Noschka and colleagues have found that the secreted small protein angiogenin and an angiogenin-derived peptide denoted as Angie 1 exhibit antimicrobial activity against *Mycobacterium tuberculosis* by inhibiting mycobacterial proliferation [38]. In the current study, we tested the effect of various Angie peptides, in silico predicted small peptide derivatives from human angiogenin, on the growth of *C. difficile *in vitro and on the cytotoxic activity of TcdA and TcdB from this bacterium. Out of six tested Angie peptides (Angie 1, 3, 5, 6, 7, and reference), the Angie peptides 1, 3, and 5 prevented the growth of *C. difficile* and reduced the cytotoxicity of the toxins. Angie 5 had the highest inhibitory potential towards the bacterium and both toxins. In the process of gaining a mechanistic understanding of the underlying toxin-inhibiting mechanism, we found that neither the binding of the toxins to the cell surface nor the glucosyltransferase activity are inhibited by the Angie peptides.

## Results

### Design and properties of Angie peptides

**Fig. 1:**
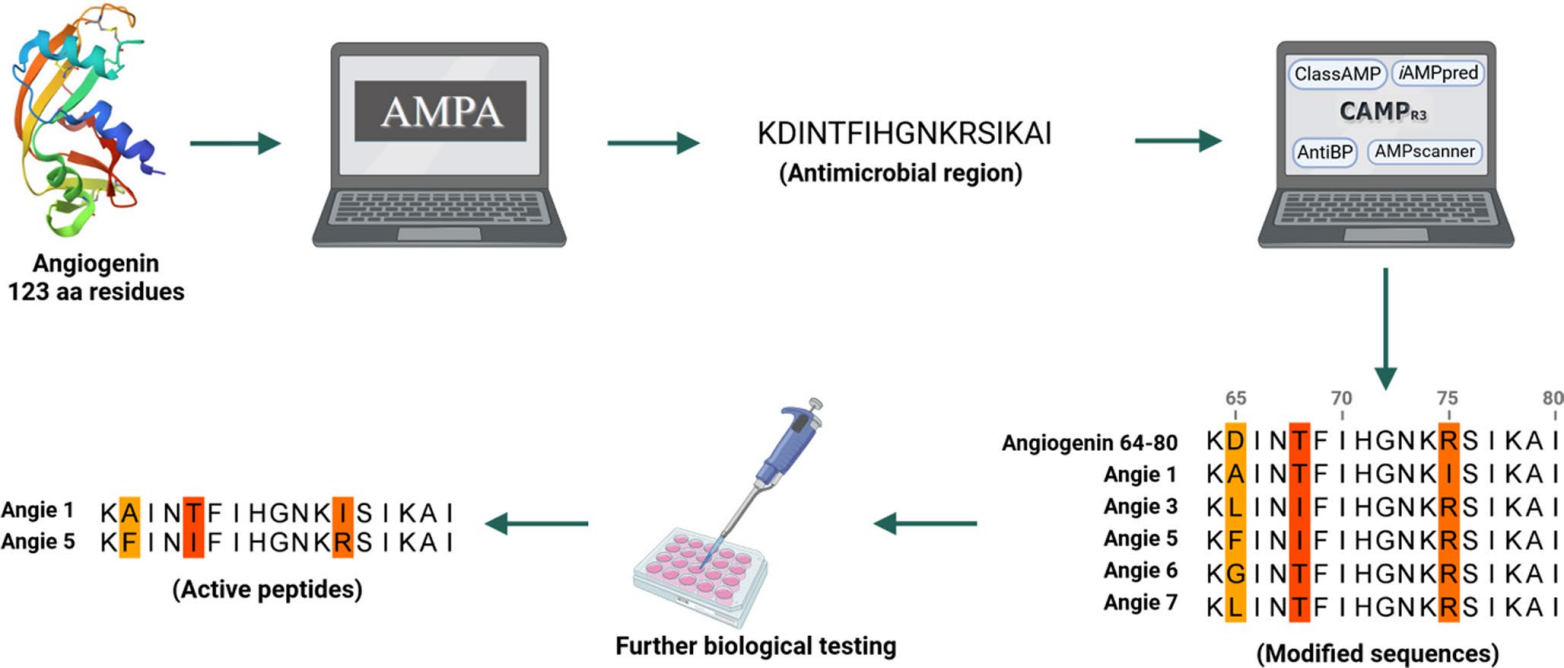
Antimicrobial prediction of Angie peptides. The antimicrobial region 64–80 was predicted by AMPA from the angiogenin precursor. Angiogenin 64–80 was optimized using CAMP_R3_ and then evaluated by CAMP_R3_, Antibp2, ClassAMP, AMP scanner, and iAMP. The original peptide and the modified variants Angie 1, 3, 5, 6, and 7 were tested, yielding Angie 1 and Angie 5 as the most active peptides. Angie 2 and 4 were not selected for synthesis. The figure was created in BioRender. Rodriguez, A. (2024) BioRender.com/d56e243

A previous study by Noschka and colleagues identified angiogenin (123 amino acids) from a human hemofiltrate library to possess antimicrobial properties against *Mycobacterium tuberculosis* [38]. Various bioinformatical tools were used to optimize and shorten human angiogenin as an antimicrobial (Fig. 1). The authors employed AMPA [49, 50] to predict the angiogenin antimicrobial region, which was determined as the aa segment comprising positions 64 to 80 of the angiogenin precursor. In the present work, the 64–80 segment was modified using the Rational Design of Antimicrobial Peptides tool of CAMP_R3_ (Waghu et al., 2015) to create several antimicrobial variants. The novel peptides and the 64–80 segment were subjected to antimicrobial prediction by using CAMP_R3_, Antibp2 [32], ClassAMP [27], Peptide AMP Scanner [51], and iAMPpred [37]. The top hits from this list were Angie 1 [38], and the new predicted 3, 5, 6, and 7 (17 amino acids), which were selected for synthesis and evaluation. The original region of angiogenin 64–80 was synthesized and termed reference Angie. The Angie peptides and the reference Angie differ only in the amino acids at positions 2, 5, and 12. Further characteristics of the Angie peptides, including name, sequence, monoisotopic mass, pI, net charge at pH 7.4, and Gravy (Grand average of hydropathicity) values, are summarized in Table 1. Since antimicrobial peptides might not only act on microbes but also on factors secreted by microbes, such as bacterial toxins, they appear to be attractive candidates for evaluation of antitoxin capabilities. As such, the Angie peptides underwent first, antimicrobial testing for *C. difficile* and second, biological testing for antitoxin activity.

**Table 1:** Properties of Angie peptides. Name, sequence, monoisotopic mass, pI, net charge at pH 7.4, and gravy (Grand average of hydropathicity) score for the tested Angie peptides

Name	Sequence	Monoisotopic mass [Da]	pI	Net charge at pH 7.4	Gravy
Angiogenin 64–80 (reference Angie)	KDINTFIHGNKRSIKAI	1954.1112	10.17	2.193	−0.541
Angie 1	KAINTFIHGNKISIKAI	1867.1044	10.17	2.192	0.3
Angie 3	KLINIFIHGNKRSIKAI	1964.2047	11.44	3.192	0.194
Angie 5	KFINIFIHGNKRSIKAI	1998.1891	11.44	3.192	0.135
Angie 6	KGINTFIHGNKRSIKAI	1896.1057	11.44	3.192	−0.359
Angie 7	KLINTFIHGNKRSIKAI	1952.1683	11.44	3.192	−0.112

### Angie 5 possesses antimicrobial activity against *C. difficile*

**Fig. 2:**
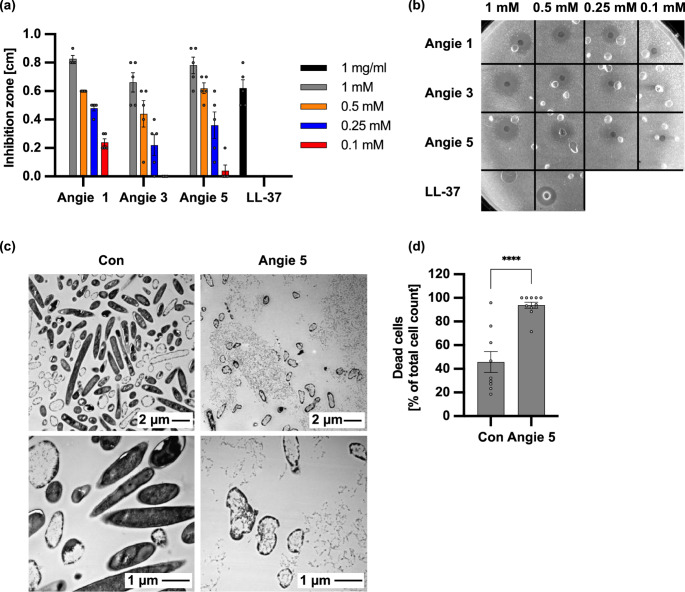
Antimicrobial activity of Angie peptides against *C. difficile*. **a** For the radial diffusion assay an agar plate was prepared with a *C. difficile* overnight culture. Wells were put in the agarose and filled with 1 mM, 0.5 mM, 0.25 mM, and 0.1 mM Angie 1, 3, and 5 Angie. The plate was incubated at 37 °C for 3 h and an overlay with BHI-Agar was conducted. After overnight incubation, inhibition zones were measured. As a positive control 1 mg/ml LL-37 was used. The inhibition zone is given in cm, mean +/- SEM (*n* = 4–5 values from four to five independent experiments). **b** Representative image for the radial diffusion assay. **c-d** *C. difficile* was grown and harvested by centrifugation, while the cells were reconstituted in 10 mM phosphate solution and either Angie 5 (0.5 mM) or respective amount of its solvent (H_2_O) was added. After incubation for 1 h at 37 °C with a sterile liquid, vaseline overlay for anaerobic conditions, the samples were fixed, postfixed, stained with uranyl acetate, embedded in epon and ultrathin sections were prepared. At least 25 pictures per sample were taken from one experiment, while overview pictures (*n* = 9–11 pictures) were used for counting of dead and alive cells and dead cells are given as percent of the total cell count, mean +/- SEM (d). Significance was tested using unpaired t test. The *p*-value for unpaired the t test is *p* < 0.0001 as indicated. (* *p* < 0.1, ** *p* < 0.01, *** *p* < 0.001, **** *p* < 0.0001, ns not significant)

A radial diffusion assay was performed to assess whether the Angie peptides possess antimicrobial activity against *C. difficile*. Here, initially higher concentrations of the Angie peptides were tested and inhibition zones around the Angie peptides and the positive control were measured (Supplementary Fig. 1). As shown in the representative image for the radial diffusion assay in Supplementary Fig. 1b, Angie 5 provided the largest inhibition zone, followed by Angie 3 and Angie 1. The antimicrobial peptide LL-37, which has been previously shown to possess activity against *C. difficile* [36], served as positive control. For Angie 6, 7, and the reference Angie peptide, no inhibition of *C. difficile* growth was observed, as only a minor or no inhibition zone was visible. Therefore, Angie 1, 3, and 5 showed antimicrobial activity against *C. difficile*, while 6, 7, and the reference Angie provided no antimicrobial capacity towards *C. difficile*. As very high concentrations were tested, a concentration series, including lower concentrations was tested for the *C. difficile* inhibiting Angie peptides, Angie 1, 3, and 5, employing the radial diffusion assay (Fig. 2a, b). Again, LL-37 served as positive control for *C. difficile* inhibition. Here, Angie 1, 3, and 5 showed a comparable and dose-dependent inhibition of *C. difficile*, except for the lowest concentration of 100 µM where Angie 1 showed the largest inhibition zone.

To further investigate the underlying mode of inhibition that the Angie peptides exhibit towards *C. difficile*, we performed transmission electron microscopy, exemplarily, using Angie 5 (Fig. 2c, d). The *C. difficile* sample treated with Angie 5 demonstrated mostly dead bacterial cells with disrupted cell membranes and cytoplasmic leakage. In the control sample where *C. difficile* was treated with the solvent (water) mostly living cells with intact cellular membranes were observed. However, also the control sample contained some damaged bacterial cells, as sample preparation cannot fully guarantee anaerobic conditions.

To test whether also other bacteria are inhibited by the Angie peptides we tested the ESKAPE pathogens, including *Pseudomonas (P.) aeruginosa*, *Acinetobacter (A.) baumannii*, *Escherichia (E.) coli*, *Enterococcus (E.) faecium*, *Staphylococcus (S.) aureus*, and *Klebsiella (K.) pneumoniae* (Supplementary Table 1), employing the radial diffusion assay. Once again, the peptide LL-37 was used as positive control of bacterial inhibition. However, from the tested pathogens only two, namely *P. aeruginosa* and *A. baumannii*, were inhibited by the Angie peptides (Supplementary Fig. 2). *P. aeruginosa* was inhibited by Angie 1, 3, and 5 in a comparable manner, while *A. baumannii* was only inhibited by Angie 5. Angie 6, 7, and the reference Angie showed no inhibition of the ESKAPE pathogens.

### Angie 5 protects HeLa, Vero, and CaCo-2 cells from intoxication with TcdB

**Fig. 3:**
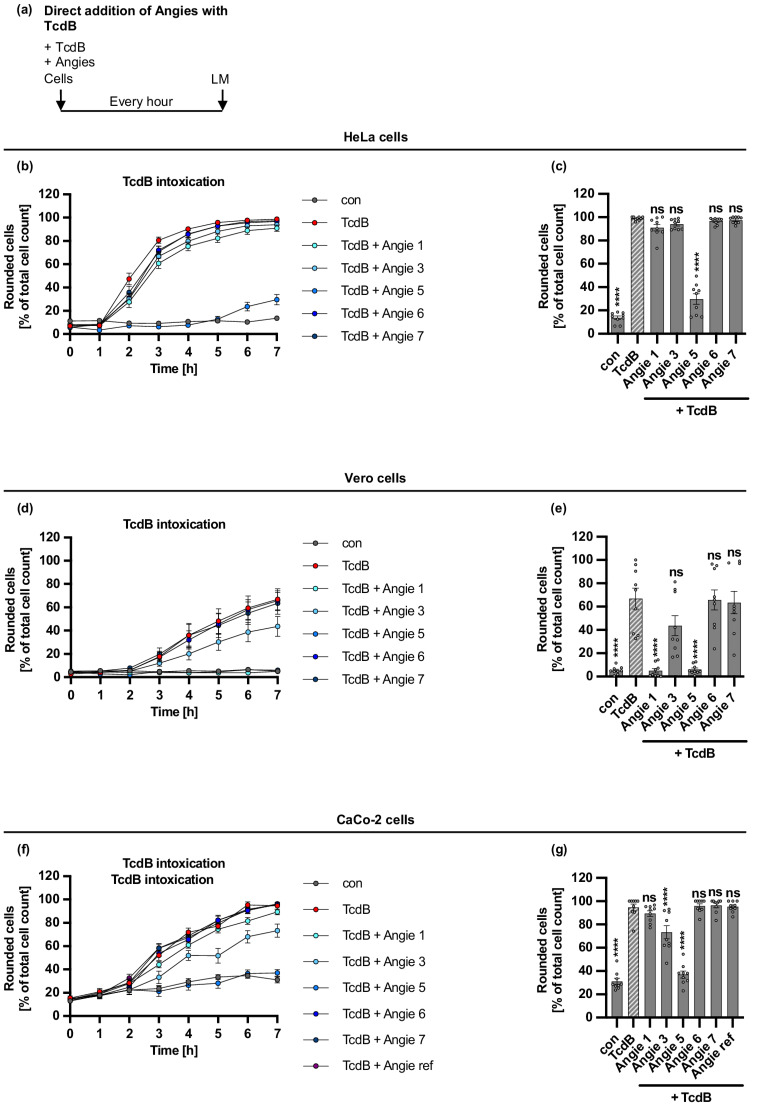
Effect of Angie peptides on TcdB-mediated cell rounding of HeLa, Vero, and CaCo-2 cells.** a** Procedure of the cell morphology assay in a schematic representation. TcdB and Angies were added simultaneously to cells and incubated for 7 h at 37 °C. Pictures were taken every hour using the light microscope (LM). **b-g** TcdB (10 pM (b-e)/200 pM (f-g)) and the different Angies (100 µM) or the respective amount of its solvent (H_2_O) were added together in FCS-free medium to HeLa cells (b-c), Vero cells (d-e) or CaCo-2 cells (f-g). The cells were incubated for 7 h at 37 °C, and pictures were taken every hour. Rounded cells are given as percent of the total cell count, mean +/- SEM (*n* = 9 values from three independent experiments, each performed with triplicates (three independent wells)), for the time course of the whole experiment (b, d, f) or for the endpoint after 7 h (c, e, g). Significance was tested using one-way ANOVA followed by Dunnett’s multiple comparison test and refers to TcdB-treated controls (TcdB) (* *p* < 0.1, ** *p* < 0.01, *** *p* < 0.001, **** *p* < 0.0001, ns not significant)

To investigate whether the Angie peptides possess antitoxin activity towards TcdB, the historical *C. difficile* strain VPI 10,463 was used. Within cells, the GTD of TcdB modifies small GTPases of the Rho and Ras-family, such as Cdc42, RhoA, and Rac1, which are regulators of the cytoskeleton. This modification causes the detrimental collapse of the cytoskeleton and ultimately leads to cell rounding. Therefore, peptides can be readily tested with reliable readout for their protective effects against TcdB-induced cell rounding by microscopic analysis of the cell morphology of intoxicated cells. To this end, HeLa, Vero, and CaCo-2 cell monolayers were treated simultaneously with the different Angie peptides and TcdB in serum-free medium to circumvent effects mediated by serum components. After an incubation at 37 °C for a time period of 7 h, microscopic images were obtained every hour. Rounded, intoxicated and flat, non-intoxicated cells were counted for each time point, while rounded cells from the total cell count are displayed in Fig. 3, for HeLa cells (Fig. 3b, c), Vero cells (Fig. 3d, e), and the physiologically more relevant human colon carcinoma cell line CaCo-2 (Fig. 3f, g). HeLa and CaCo-2 cells were only protected by Angie 5, whereas Vero cells were additionally protected by Angie 1 and 3. Angie 6 and 7 did not inhibit TcdB-mediated cell rounding in any of the three tested cell lines. Supplementary Fig. 3 shows representative images for HeLa, Vero, and CaCo-2 cells after 7 h of treatment with TcdB and Angie peptides. Moreover, the reference Angie peptide did not inhibit TcdB-mediated cell rounding in the two tested cell lines, Vero (Supplementary Fig. 4) and CaCo-2.

**Fig. 4:**
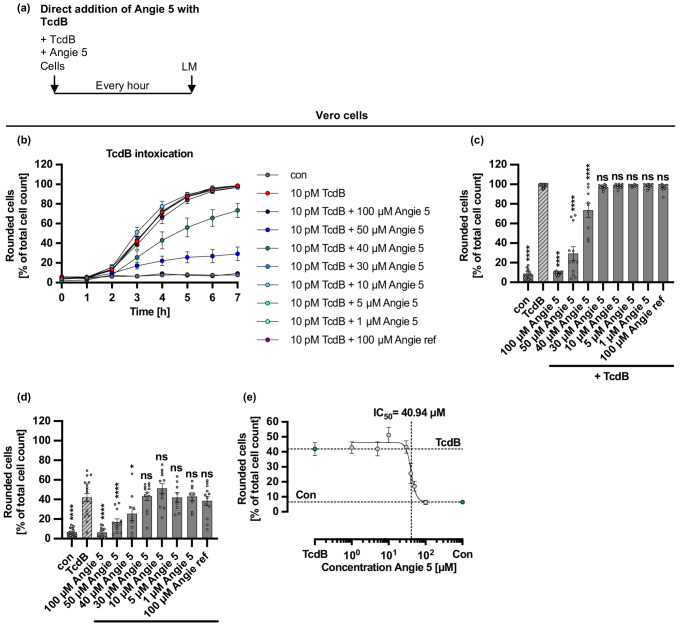
Effect of Angie 5 on TcdB-mediated cell rounding of Vero cells. **a** Procedure of the cell morphology assay in a schematic representation. TcdB and Angies were added simultaneously to cells and incubated for 7 h at 37 °C. Pictures were taken every hour using the light microscope (LM). **b-e** TcdB (10 pM (b-e)) and the different concentrations of Angie 5 and the reference Angie (100 µM) or the respective amount of its solvent (H_2_O) were added together in FCS-free medium to Vero cells (b-e). The cells were incubated for 7 h at 37 °C, and pictures were taken every hour. Rounded cells are given as percent of the total cell count, mean +/- SEM (*n* = 9–18 values from six independent experiments, each performed with triplicates (three independent wells)), for the time course of the whole experiment (b), after 3 h (d-e) for the endpoint after 7 h (c). **(e)** The IC_50_ values for Angie 5 were calculated from the percentage of rounded cells of the total cell count after 3 h of incubation. A nonlinear regression model with variable slope (GraphPad Prism, log(inhibitor) versus response (variable slope, four parameters)) was fitted to values, and IC_50_ values were given based on the fit. Significance was tested using one-way ANOVA followed by Dunnett’s multiple comparison test and refers to TcdB-treated controls (TcdB) (* *p* < 0.1, ** *p* < 0.01, *** *p* < 0.001, **** *p* < 0.0001, ns not significant)

Since the peptide Angie 5 showed the most robust inhibition of TcdB-mediated cell rounding in the three investigated cell lines, HeLa, Vero, and CaCo-2 cells, also lower concentrations of Angie 5 were analyzed for their effect on TcdB-mediated cell rounding in Vero cells (Fig. 4). In addition to 100 µM Angie 5, also 50 µM and 40 µM of Angie 5 showed inhibition of TcdB-mediated cell rounding. However, the next lower concentration of Angie 5 (30 µM) showed no significant inhibition of TcdB-mediated cell rounding. For the time point 3 h after addition of the components, the half-maximal inhibitory concentration (IC_50_) value was calculated for Angie 5 which resulted in an IC_50_ of 40.94 µM ± 1.08 µM (± SEM) (Fig. 4d-e). To investigate whether the inhibitory effect of Angie 5 can be enhanced, TcdB and different concentrations of Angie 5 were preincubated for 15 min at room temperature in FCS-free medium, before addition to Vero cells (Supplementary Fig. 5). Similarly, as before the IC_50_ value was calculated for Angie 5 after three hours of TcdB intoxication which resulted in an IC_50_ of 46.34 µM ± 1.14 µM (± SEM) (Supplementary Fig. 5d-e). To compare whether the preincubation of TcdB with Angie 5 had an effect on inhibition of Angie 5, normalized values after 3 h of intoxication were compared (Supplementary Fig. 6). However, for all tested concentrations except for 10 µM Angie 5, there was no significant difference in rounded cells, resulting in no enhanced inhibition of TcdB-mediated cell rounding by preincubation of toxin and inhibitor for most of the tested concentrations.

### Angie 5 protects cells from intoxication of Vero cells with TcdA and the medically relevant combination of TcdA and TcdB

**Fig. 5:**
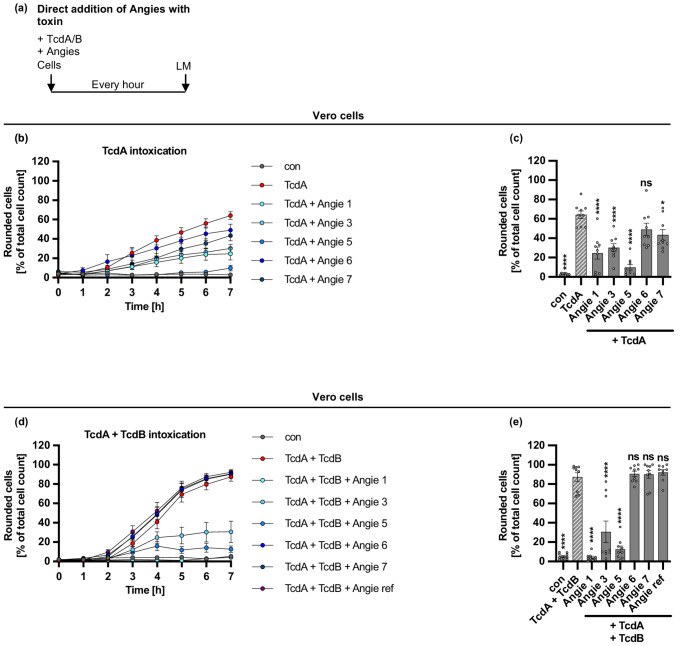
Effect of Angie peptides on TcdA- and the combination of TcdA- and TcdB-mediated cell rounding of Vero cells.** a** Procedure of the cell morphology assay in a schematic representation. Toxin and Angies were added simultaneously to cells and incubated for 7 h at 37 °C. Pictures were taken every hour using the light microscope (LM). **b-e** TcdA (180 pM) (b, c) or TcdA (180 pM) with TcdB (10 pM) (d, e) and the different Angies (100 µM) or the respective amount of its solvent (H_2_O) were added together in FCS-free medium to Vero cells. The cells were incubated for 7 h at 37 °C, and pictures were taken every hour. Rounded cells are given as percent of the total cell count, mean +/- SEM (*n* = 9 values from three independent experiments), for the time course of the whole experiment (b, d) or for the endpoint after 7 h (c, e). Significance was tested using one-way ANOVA followed by Dunnett’s multiple comparison test and refers to TcdB-treated controls (TcdB) (* *p* < 0.1, ** *p* < 0.01, *** *p* < 0.001, **** *p* < 0.0001, ns not significant)

Next, we examined whether the Angie peptides possess protective capabilities against another clinically relevant toxin produced by *C. difficile*, namely TcdA. As described above for TcdB, Vero cells were treated with the Angie peptides and TcdA. Subsequently, TcdA-induced cell rounding was examined via microscopy. The results on cell rounding caused by the treatment of Vero cells with TcdA in the presence of Angie peptides in comparison to the control (without Angie peptides) are depicted in Fig. 5, while representative cell images are shown in Supplementary Fig. 7. Here, inhibition of TcdA-induced cell rounding was strongest for Angie 5 followed by Angie 1 and 3.

Moreover, the clinically relevant combination of TcdA and TcdB was investigated in a comparable manner using Vero cells (Fig. 5, Supplementary Fig. 7). The combination of TcdA and TcdB was also inhibited by Angie 5 and, to a lesser extent, by Angie 1 and 3. In Supplementary Fig. 7, representative images are shown for treatment of Vero cells with Angie peptides and TcdA or the combination of TcdA and TcdB after 7 h. Previous studies have shown that TcdB is more cytotoxic than TcdA, and additionally, TcdB is regarded to be the major virulence factor of *C. difficile*. Thus, for the subsequent performed experiments, only TcdB was investigated [9, 28].

### Angie 5 inhibits TcdB-mediated glucosylation of Rac1 in Vero cells in a time- and concentration-dependent manner

**Fig. 6:**
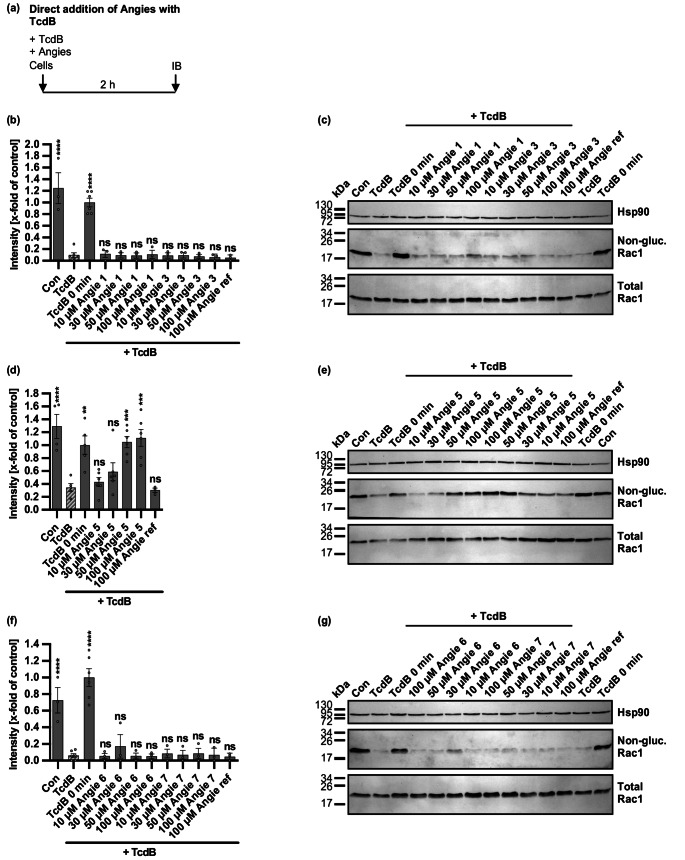
Effect of Angie peptides on glucosylation status of intracellular Rac1 in TcdB-treated Vero cells. **a** Procedure of the assay analyzing the glucosylation status of intracellular Rac1 in a schematic representation. TcdB and Angies were added simultaneously to cells and incubated for 2 h at 37 °C. Afterwards, cell lysates were generated and subjected to immunoblotting (IB). **b-g** Vero cells were treated with different concentrations of the Angie peptides or the respective amount of solvent (H_2_O) and TcdB (50 pM) in FCS-free medium for 2 h at 37 °C. As controls, cells were either not treated with TcdB (con) or cell lysates were generated directly after TcdB addition (TcdB 0 min). Whole-cell lysates were generated and processed by SDS-PAGE and immunoblotting. Then, signals for non-glucosylated Rac1, total Rac1, and Hsp90 were detected, while representative images of immunoblots are shown (c, e, g). Afterwards, signal intensity was determined, and signals for non-glucosylated Rac1 were normalized to the loading control Hsp90 or Ponceau-S staining, and the control 0 min TcdB and values are given as mean +/- SEM (*n* = 3–6 values from three independent experiments) (b, d, f). Significance was tested using one-way ANOVA followed by Dunnett’s multiple comparison test and refers to TcdB-treated controls (TcdB) (* *p* < 0.1, ** *p* < 0.01, *** *p* < 0.001, **** *p* < 0.0001, ns not significant)

Given that intoxication of cells with TcdA and TcdB was inhibited by selected Angie peptides, we analyzed exemplarily with TcdB the inhibitory effect of the peptides on the glucosylation of intracellular Rac1 upon uptake of the toxin into Vero cells. To this end, Vero cells were treated with the different concentrations of the Angie peptides and TcdB, followed by immunoblotting for the detection of unmodified, non-glucosylated Rac1, as well as total Rac1, and Hsp90 as loading controls (Fig. 6). Whole-cell lysates from non-treated control cells exhibit a strong signal in the immunoblot with the antibody recognizing only non-glucosylated Rac1, indicating that intracellular Rac1 proteins were not glucosylated in these samples. However, when cells were only treated with TcdB for 2 h, almost no or a very weak signal was observed in the immunoblot, indicating that most of the intracellular Rac1 was glucosylated and thus modified by the toxin. As expected, almost no decrease of the Rac1 signal was observed, when cell lysates were analyzed from TcdB-treated cells immediately after toxin addition (time point 0 min), confirming that residual TcdB during in vitro sample preparation did not cause glucosylation of Rac1 within the whole-cell lysates. For all concentrations of Angie 1, 3, 6, 7, and the reference Angie no signal for Rac1 was obtained in the immunoblot with the antibody recognizing only non-glucosylated Rac1, indicating no inhibition of TcdB-induced Rac1 glucosylation by those peptides (Fig. 6b-c, e-f). In contrast, a concentration-dependent increase in signal for non-glucosylated Rac1 was observed for Angie 5, indicating inhibition of TcdB-mediated Rac1 glucosylation by this peptide (Fig. 6d-e).

**Fig. 7:**
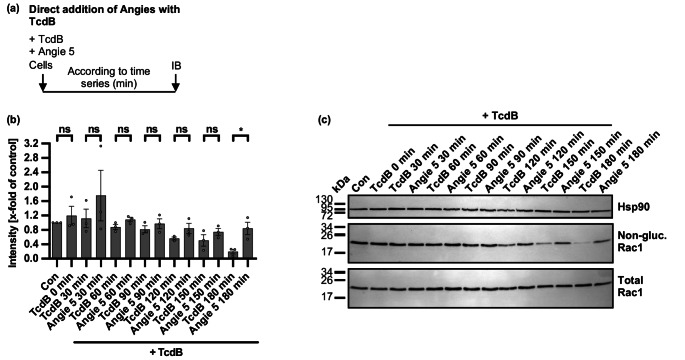
Time-dependent effects of Angie 5 on glucosylation status of intracellular Rac1 in TcdB-treated Vero cells.** a** Procedure of the assay analyzing the glucosylation status of intracellular Rac1 in a schematic representation. TcdB and Angie 5 were added simultaneously to cells and incubated for increasing time intervals at 37 °C. After wards, whole-cell lysates were generated and subjected to immunoblotting (IB). **b-c** Vero cells were treated with 100 µM of Angie 5 or the respective amount of solvent (H_2_O) and TcdB (50 pM) in FCS-free medium for 30 min, 60 min, 90, min, 120 min, 150–180 min at 37 °C. For control, cells were not treated with TcdB or 0 min before generation of cell lysates. After treatment, cell lysates were generated and processed by SDS-PAGE and immunoblotting. Then, signals for non-glucosylated Rac1, total Rac1, and Hsp90 were detected, while representative images of immunoblots are shown (c). Afterwards, signal intensity was determined and signals for non-glucosylated Rac1 were normalized to the loading control Hsp90 and the non-treated control (Con), while values are given as mean +/- SEM (*n* = 3 values from three independent experiments) (b). Significance was tested using unpaired t test (* *p* < 0.0332, ** *p* < 0.0021, *** *p* < 0.0002, **** *p* < 0.0001, ns not significant)

For a closer investigation of the TcdB inhibition by Angie 5, a time-lapse intoxication experiment was performed with a subsequent analysis of the Rac1 glucosylation status via immunoblotting. As such, Vero cells were either treated with 100 µM Angie 5 and TcdB or only with TcdB for increasing time intervals and up to 3 h (Fig. 7). As a result, the present Angie 5 clearly resulted in the delay of TcdB-mediated Rac1 glucosylation.

### Angie 5 inhibits TcdB-induced collapse of the actin cytoskeleton in Vero cells

**Fig. 8:**
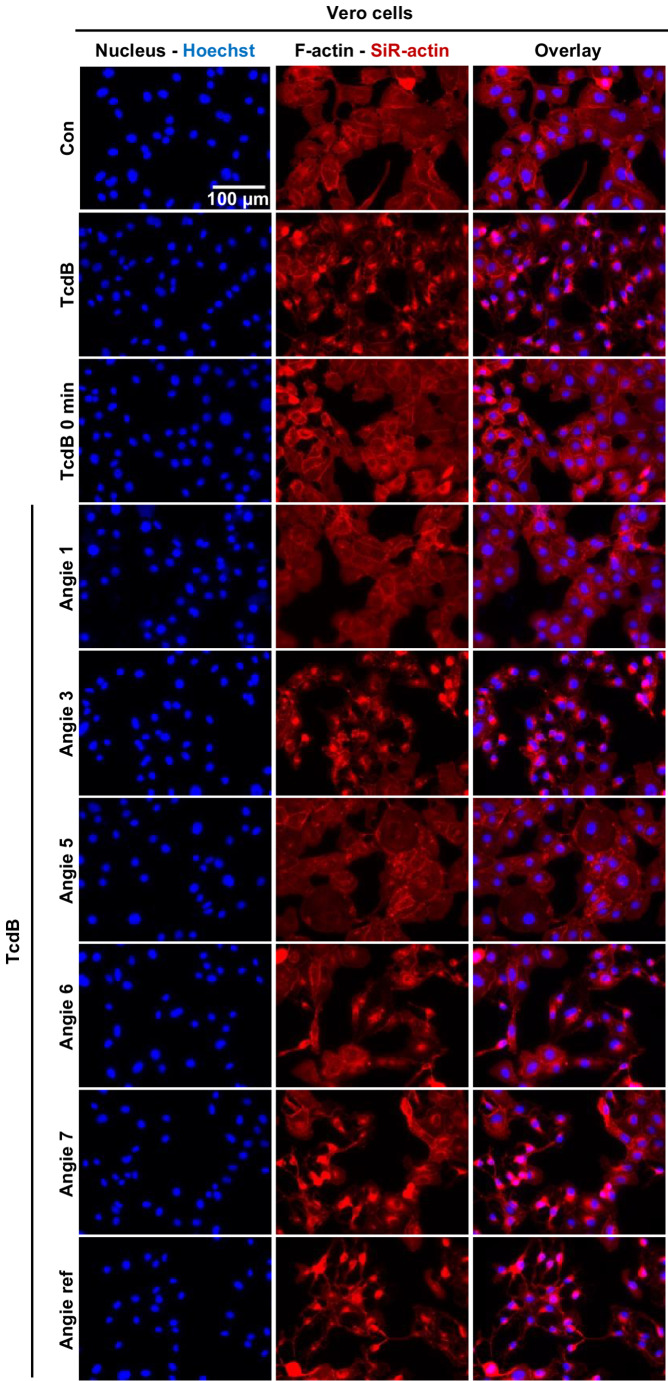
Effect of Angie peptides on F-actin in TcdB intoxicated Vero cells. Vero cells were treated with various Angie peptides (100 µM) as indicated or with the respective amount of solvent (H_2_O) and TcdB (50 pM) in FCS-free medium for 2 h at 37 °C. As control, cells were either not treated with TcdB or fixed immediately after TcdB addition (TcdB 0 min). Next, cells were permeabilized, and quenching was performed. Cell nuclei were stained using Hoechst 33,342, while F-actin was stained using SiR-actin. Representative images are shown from a single experiment (*n* = 45 from three independent experiments)

To further confirm our findings that Angie 5 can inhibit TcdB intoxication of Vero cells, we implemented fluorescence microscopy to analyze the status of the actin cytoskeleton after TcdB intoxication. Therefore, Vero cells were treated with the Angie peptides and TcdB or only with TcdB, followed by cell nuclei staining with Hoechst 33,342 and F-actin staining with the highly specific F-actin probe SiR-actin. Upon intoxication of cells with TcdB, F-actin filaments appeared condensed, while in untreated control cells, F-actin filaments were present in an organized, regular structure (Fig. 8). When cells were treated with Angie 1 and 5 and intoxicated with TcdB, the F-actin structure appeared more comparable to untreated control cells. In contrast, Angie 3, 6, 7 and the reference peptide did not inhibit TcdB-mediated collapse of the actin cytoskeleton.

### Angie 5 does not lead to precipitation of TcdB in vitro

**Fig. 9:**
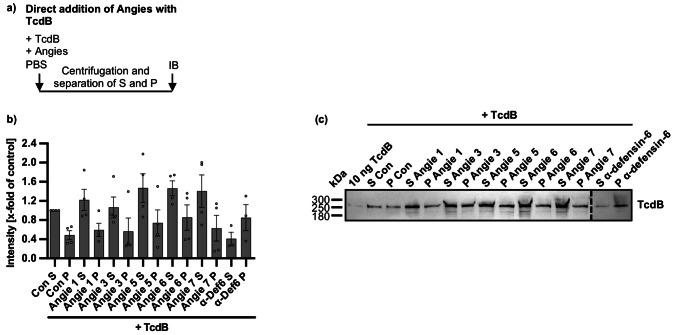
Effect of Angie peptides on the precipitation of TcdB in vitro. **a** Procedure of the precipitation assay in a schematic representation. Angie peptides and TcdB were mixed, centrifuged, and samples were prepared for analysis via immunoblot (IB) **b c** The different Angie peptides (100 µM) or the respective amount of solvent (H_2_O) and TcdB (50 ng) were incubated in PBS (total volume 25 µl) for 30 min at 37 °C. Afterwards, centrifugation was performed, and the supernatant and pellet fraction were separated. Samples were prepared for SDS-PAGE and immunoblot, while signals for TcdB were detected and quantified. Afterwards, signal intensity for TcdB was normalized to the supernatant fraction of the TcdB-treated control (Con S), while values are given as mean +/- SEM (*n* = 3–4 values from four independent experiments) (b). A representative experiment is shown (c). Significance was tested using one-way ANOVA followed by Dunnett’s multiple comparison test and refers to TcdB-treated controls (TcdB) (* *p* < 0.1, ** *p* < 0.01, *** *p* < 0.001, **** *p* < 0.0001, ns not significant)

So far, the most prominent and robust inhibition of TcdB was mediated by Angie 5. To further investigate the underlying mechanism of inhibition, we tested whether Angie 5 or the other Angie peptides form precipitates with TcdB. In such scenario, this would lead to entrapment of TcdB in toxin-peptide complexes in solution, thereby preventing cell entry of the toxin. Interestingly, this inhibitory mechanism was already shown by our laboratory for the human peptide α-defensin-6 against TcdB [6]. Therefore, TcdB and the Angie peptides, or as positive control, α-defensin-6 were co-incubated, and next, the supernatant and possible pellet fraction were separated via centrifugation. Samples were further processed using SDS-PAGE and immunoblotting while signals for TcdB were detected with a TcdB-specific antibody. The co-incubation of TcdB with α-defensin-6 served as a positive control for the precipitation of TcdB. In this sample, most of the signal for TcdB was obtained in the pellet fraction, indicating precipitate formation of α-defensin-6 with TcdB (Fig. 9). In the negative control (TcdB alone), most of the signal obtained for TcdB was found in the supernatant fraction. Importantly, for all Angie peptides, the signal for TcdB is strongest in the supernatant fraction, indicating no precipitate formation of the Angie peptides with TcdB.

### Angie 5 does not prevent binding of TcdB to Vero cells

**Fig. 10:**
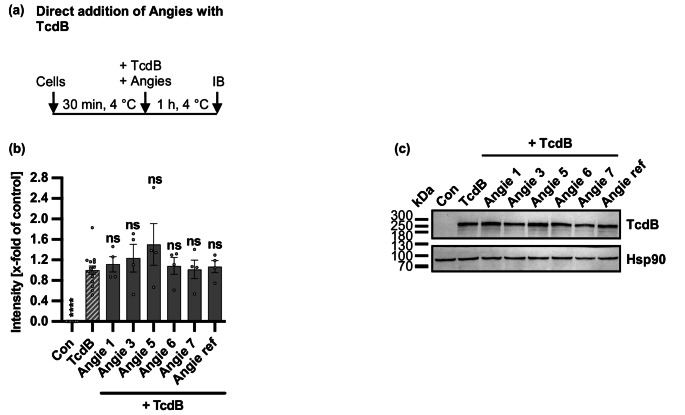
Effect of Angie peptides TcdB binding to Vero cells. **a** Procedure of the immunoblot-based binding assay in a schematic representation. First, cells were precooled at 4 °C and Angie peptides and TcdB were added. Then, samples were prepared for analysis via immunoblotting against TcdB (IB). **(b-c)** Vero cells were incubated for 30 min at 4 °C. Then, different Angie peptides (100 µM) or the respective amount of solvent (H_2_O) and TcdB (500 pM) were added to precooled cells and incubated for further 60 min at 4 °C to allow toxin binding but prevent internalization. After that, whole-cell lysates were prepared for SDS-PAGE and immunoblotting against TcdB and Hsp90. Afterwards, signal intensity for TcdB was normalized to the loading control Hsp90 or Ponceau-S staining and the TcdB-treated control (TcdB), while values are given as mean +/- SEM (*n* = 4 values from four independent experiments) (b). A representative experiment is shown (c). Significance was tested using one-way ANOVA followed by Dunnett’s multiple comparison test and refers to TcdB-treated controls (TcdB) (* *p* < 0.1, ** *p* < 0.01, *** *p* < 0.001, **** *p* < 0.0001, ns not significant)

Since no precipitate formation of the Angie peptides and TcdB was observed, the underlying mechanism of inhibition might be related to subsequent steps during the uptake process of TcdB into target cells. As such, we investigated the first step of intoxication, the binding of TcdB to the cell surface of TcdB. Therefore, an immunoblot-based binding assay was performed where Vero cells were treated with Angie peptides and TcdB at 4 °C to allow toxin binding but where endocytosis is prevented (Fig. 10). Subsequently, cell surface-bound TcdB was analyzed via immunoblotting of whole-cell lysates and by using an antibody specifically recognizing TcdB. With this approach, we observed that all Angie peptides had no negative influence on TcdB binding to Vero cells, while Angie 5 might cause a slight, not significant enhancement in toxin binding to cells.

### Angie 5 does not inhibit glucosyltransferase activity of TcdB in vitro

**Fig. 11:**
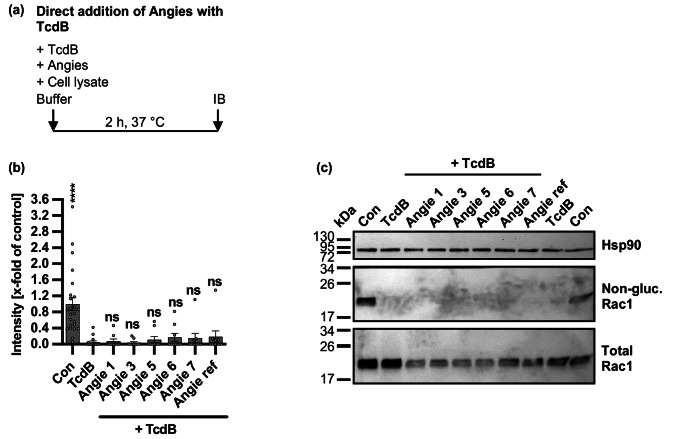
Effect of Angie peptides on enzyme activity of TcdB in vitro using CaCo-2 cell lysate.** a** Procedure of the enzyme activity assay analyzing the glucosylation status of Rac1 in vitro in a schematic representation. The different Angie peptides or the respective amount of solvent (H_2_O) and TcdB were added directly to CaCo-2 cell lysate. Then the samples were incubated for 2 h at 37 °C and subsequently analyzed via SDS-PAGE and immunoblotting. **(b-c)** The different Angie peptides (100 µM) or the respective amount of solvent (H_2_O) and TcdB (10 nM) were added directly to CaCo-2 cell lysate (40 µg). After that samples were prepared and analyzed via SDS-PAGE and immunoblotting, while signals for non-glucosylated Rac1, total Rac1, and Hsp90 as loading control were detected and quantified. Values are given as mean +/- SEM (*n* = 5–24 values from five independent experiments) (b). Blot pictures for representative experiments are shown (c). Significance was tested using one-way ANOVA followed by Dunnett’s multiple comparison test and refers to TcdB-treated controls (TcdB) (* *p* < 0.1, ** *p* < 0.01, *** *p* < 0.001, **** *p* < 0.0001, ns not significant)

Next, we investigated whether the glucosyltransferase activity of TcdB is inhibited by the Angie peptides in an in vitro immunoblot-based enzyme activity assay. Therefore, Angie peptides and TcdB were incubated together with CaCo-2 cell lysate, serving as source for a substrate target of TcdB, such as Rac1. Then, samples were subjected to SDS-PAGE and immunoblotting against non-glucosylated Rac1 and Hsp90 (Fig. 11). Without TcdB being present in the cell lysate (con), as expected, non-glucosylated Rac1 was recognized by an antibody specific for non-glucosylated Rac1. In contrast, when TcdB was added to the cell lysate, glucosylation of Rac1 by TcdB strongly decreased the antibody detection of non-glucosylated Rac1. When the Angie peptides were co-added with TcdB to the cell lysate, Rac1 antibody signals were still reduced, indicating that the Angie peptides did not inhibit the glucosyltransferase activity of TcdB in vitro (Fig. 11c-d). Since the glucosylation buffer used for the determination of the glucosyltransferase activity of TcdB in vitro contains BSA, there was a possibility of the Angie peptides binding to the BSA within the buffer. Therefore, the glucosylation buffer was produced without BSA and the influence of the Angie peptides on the enzyme activity of TcdB was assessed again, using CaCo-2 cell lysate generated in glucosylation buffer without BSA. Here, comparable to the results obtained with BSA in the glucosylation buffer, no inhibition of the enzyme activity of TcdB by the Angie peptides was observed (Supplementary Fig. 8). In addition, the effect of the Angie peptides on TcdB-mediated cell rounding in Vero cells, employing the cytopathic cell rounding assay was assessed, but this time using serum containing conditions (Supplementary Fig. 9). Also, under these conditions, the Angie peptides 1, 3, and 5 showed inhibition of TcdB-mediated cell rounding of Vero cells. Taken together, BSA and/or other serum proteins seem not to capture Angie peptides and thus do not interfere with our experiments.

### In silico prediction of the complex between TcdB and Angie 5

Despite the rapid advancement of bioinformatics tools for predicting peptide-protein complexes, this task remains highly challenging today [55]. This is especially true for long peptides, as their structural flexibility and complex interactions with proteins make accurate modeling more difficult. Additionally, the use of large proteins as receptors significantly increases the search space for the peptide, further complicating efficient prediction. To address these challenges, we employed three different search algorithms implemented in the servers AlphaFold3 [1], HPEPDOCK [57], and PEP-SiteFinder [44]. This approach enabled us to explore the full conformational space of Angie 5 while ensuring a thorough search of its interaction region with the receptor.

**Fig. 12:**
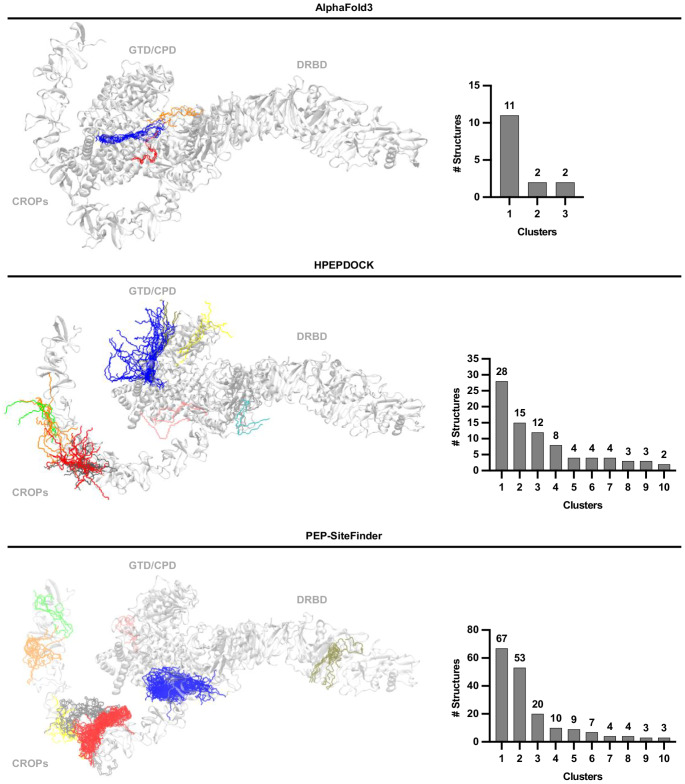
Clusters obtained from each protein-peptide docking software. TcdB is shown as a transparent gray ribbon, while peptides are represented as colored lines. Each color corresponds to a distinct cluster, defined by an RMSD threshold of 20 Å. The accompanying graph illustrates the number of structures obtained for each cluster

A total of 315 TcdB-Angie 5 complexes were obtained: 15 using AlphaFold3, 100 with HPEPDOCK, and 200 with PEP-SiteFinder. These were clustered into 23 representative structures: 3 from AlphaFold3 and 10 from each of the other two methods. This clusters show a high representativity in the regions encompassing the N-terminal glucosyltransferase domain (GTD) and the cysteine protease domain (CPD), followed by the C-terminal combined repetitive oligopeptides (CROPs). However, no significant number of clusters were found in the central delivery and receptor-binding domain (DRBD) indicating low probability of interaction by this region (Fig. 12).

Each docking software has its own scoring function, which ranks the predicted binding poses from best to worst. However, these scoring functions are not directly comparable across different software. Therefore, to select the best complex, it is necessary to normalize the binding energy using an additional server that recalculates the binding energy for all structures using a unified scoring function. Accordingly, after selecting the representative structure from each cluster based on the scoring function of its respective server, the binding energy of each structure was recalculated using the Prodigy server. This analysis revealed that the most stable TcdB-Angie 5 complex corresponded to cluster 1 from AlphaFold3 (Supplementary Fig. 10).

**Fig. 13:**
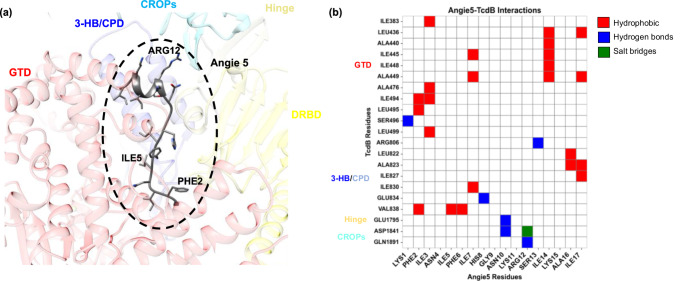
Interactions between Angie5 and TcdB. **a** Structural representation of the most stable TcdB-Angie 5 complex. Both the toxin and peptide are shown in their respective secondary structures. TcdB domains are color-coded following the same scheme as in [12]: red for GTD, blue for 3-HB/CPD, cyan for CROPs, yellow for DRBD, and gold for the hinge region. **b** Detailed interaction map between Angie 5 and TcdB. TcdB residues are shaded according to their respective domain colors. Hydrophobic interactions, hydrogen bonds, and salt bridges are represented by red, blue, and green squares, respectively

The peptide is located in a region where all domains structurally converge, despite being separated in the primary sequence (Fig. 13a). The N-terminus of Angie 5 (LYS1–GLY9) adopts an extended conformation that buries itself into a hydrophobic pocket formed at the interface between the GTD, CPD, and DRBD domains. Due to the hydrophobic nature of this region in Angie 5, the system is stabilized by a high number of hydrophobic interactions (Fig. 13b), while remaining shielded from the polar solvent. Conversely, the C-terminus (ASN10–ILE17) has a more polar nature and is located at the interface between the solvent and the protein. Accordingly, this region adopts an α-helical structure, allowing the peptide to expose its polar residues to the solvent while keeping the non-polar residues in contact with the protein (Fig. 13a). Here, the peptide establishes both electrostatic and hydrophobic interactions, with ARG12 playing a particularly important role by forming hydrogen bonds and salt bridges, which are highly stabilizing interactions. Notably, Angie 5 forms extensive interactions with the three-helical bundle (residues 766–841, referred to as 3-HB) and the hinge region (residues 1792–1834). The hinge directly interacts with a three-stranded β-sheet in the CPD (residues 742–765, termed the β-flap), which is crucial for CPD activation, as well as with 3-HB [12]. These results suggest that Angie 5 may interfere with CPD activation, potentially inhibiting the intoxication of cells caused by TcdB. In an in vitro setting the auto-processing of TcdB was not influenced by the Angie peptides (Supplementary Fig. 11).

To further validate our findings, we generated in silico mutants by modifying the Angie 5 peptide within the same structural framework, transforming it into the other peptides included in this study. The binding energy of these modified complexes was then recalculated using the same scoring approach. Consistent with the experimental results, Angie 5 and Angie 3 exhibited similar binding energies, while the other peptide variants showed lower affinities for the receptor (Supplementary Table 2, Supplementary Fig. 12). The primary difference between Angie 5 and Angie 3 is the presence of isoleucine (ILE) at position 5, whereas the other peptides have threonine (THR) in this position (Table 1). This substitution may play a key role in stabilizing the interaction, contributing to the higher binding affinity observed for Angie 5 and Angie 3.

### Determination of the stability of Angie 5 in human plasma

**Fig. 14:**
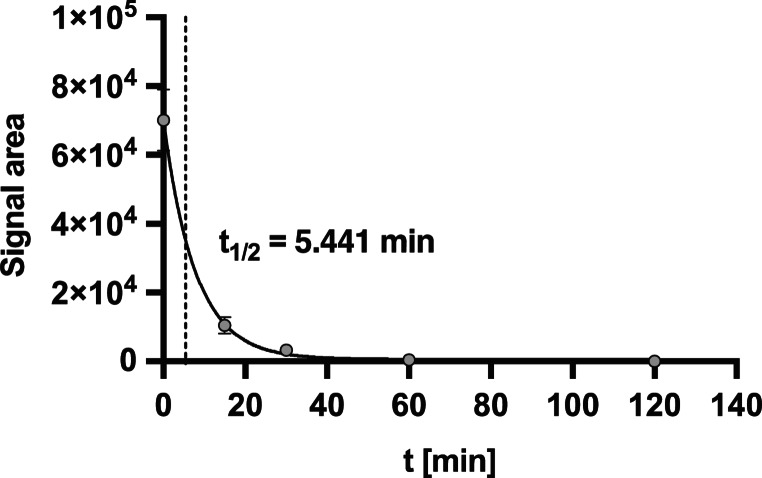
Half-life determination of Angie 5 in human plasma. Values are given as mean +/- SD (*n* = 3 values per time point). One-phase decay curve of signal area vs. time: Y = (Y_0_ - Plateau) *exp(-K*X) + Plateau, being Y_0_ = 70,043, Plateau = 642.4, and K = 0.1274

For the application and development of Angie 5 as a therapeutic agent for the treatment of CDIs in the future, the stability of the peptide is of importance and major concern. For the determination of the stability of Angie 5, its half-life was determined in human plasma using MALDI-TOF. The calculated half-life of Angie 5 in human plasma was 5.441 min (Fig. 14).

## Discussion

For the treatment of many infectious diseases, antibacterial-based therapeutic strategies, including antibiotics, are often limited or exhausted, due to an overall increase of antimicrobial resistance. This is also true for the treatment of CDIs where antibiotics used in the clinic are limited to metronidazole, vancomycin, and fidaxomicin. Moreover, therapy of CDIs is often complicated by the failure to eradicate the bacteria and the frequent recurring infections. As such, to broaden the therapeutic spectrum, there is a necessity for novel therapeutic approaches, especially anti-toxin-based approaches, which aim to target the toxins produced by pathogenic bacteria. Huge potential for novel treatment options against bacterial toxins lies within antimicrobial peptides or endogenous peptides. Recently, we identified via screening of a human-derived hemofiltrate protein library the endogenous protein α_1_-antitrypsin as a potent inhibitor against several bacterial toxins, including *Bordetella pertussis* pertussis toxin, *Clostridium botulinum* C2 toxin, *Corynebacterium diphtheriae* diphtheria toxin, and *Bacillus anthracis* fusion toxin (anthrax binding domain combined with enzyme domain from e.g., TcdB) [33, 34]. Moreover, we have shown that the human antimicrobial peptide α-defensin-1 inhibits TcdA, TcdB, and CDT [14], while α-defensin-6 possesses protective effects in cells and zebrafish embryos towards TcdA, TcdB, and the combination of both toxins [6]. This demonstrates the potential of antimicrobial peptides and endogenous proteins to fulfill more functions, including antitoxin defense.

A previous study has identified angiogenin from screening of a human hemofiltrate library to be a potent and robust inhibitor of extracellular *Mycobacterium tuberculosis* [38]. Human angiogenin (ANG or RNASE5) is a ribonuclease involved e.g., in vascularization during angiogenesis, the cleavage of tRNA, and the stimulation of ribosomal RNA synthesis. Employing computational analysis, they identified the hypothetical antimicrobial active region within angiogenin and optimized the lytic activity by amino acid exchanges to generate of a short peptide called Angie 1. Also, Angie 1 was shown to be a potent inhibitor against extra- and intracellular *Mycobacterium tuberculosis* and other bacteria that grow faster than *Mycobacterium tuberculosis*. Thus, testing of proteins and peptides with antimicrobial properties appears promising also for bacterial toxins. Therefore, in this study, we examined the inhibitory capacities of the previously identified antimicrobial Angie peptides, including the Angie 1 as mentioned earlier, towards the bacterial toxins TcdA and TcdB from *C. difficile.* The Angie peptides presented here are modified peptides derived from a short region of the endogenous protein angiogenin. Compared to the reference Angie, which is the 17 amino acid sequence naturally occurring in angiogenin, the other Angie peptides differ only by one or two amino acids at positions 2 and 5 or 12.

Our results demonstrate that Angie 5 provides the highest inhibitory potential compared to the other tested Angie peptides (Angie 1, 3, 6, 7, and the reference Angie). However, in Vero cells, Angie 1 and 3 also showed inhibition of intoxication, possibly due to different toxin-receptor dependencies and endocytic uptake rates between human and non-human cell lines. Further studies are required to examine the mode of action of Angie 5 in more detail and to unravel how the peptide inhibits TcdB. Our findings suggest that neither the binding of TcdB to cells nor the enzyme activity of TcdB is inhibited by the Angie peptides. Moreover, TcdB does not form precipitates with the Angie peptides. Further experiments will show whether e.g., the intracellular trafficking of TcdB is inhibited by Angie 5.

Small changes in the composition of the amino acids, meaning changing single residues, drastically influenced the inhibitory capacities of the Angie peptides. A higher number of hydrophobic residues, which is reflected as higher Gravy values of the Angie peptides (Table 1), improved the activity of these peptides. As such, Angie peptides that show weak to strong inhibition (Angie 1, 3, and 5) have a positive Gravy score, while Angie peptides that do not show inhibition (Angie 6, and 7, as well as the reference Angie) have a negative Gravy score. Particularly, the change of single amino acids at positions 2, 5, and 12 suggests that the inhibitory properties seem strongly connected to the hydrophobic attributes of those amino acids. Angie 1 and 3 bear the amino acids isoleucine and leucine, with hydrophobic side chains at positions 2, 5 or 12. On the other hand, isoleucine and phenylalanine residues in position 2 and 5 appear to be highly relevant for the inhibitory potential of Angie 5. As can be observed, the only difference between Angie 3 and Angie 5 is the presence of the aromatic residue Phe in position 2 of the latter, which confers it a higher activity in comparison with Angie 3. This fact indicates that the aromatic Phe residue is more relevant than the aliphatic residue Leu in these peptides. Aromatic rings are frequently involved in π-π, cation-π, and CH-π interactions, which are often crucial for protein structure and protein-ligand binding [23, 46]. In the case of π-π interactions, some aromatic residues from the toxin could be involved in the interaction with Angie 5, which would be interesting to study in the future. The findings presented in this study might also serve as a basis for further optimization of the Angie peptides, particularly Angie 5, for instance, by incorporating more aromatic residues in its sequence.

In line with the results on the importance of hydrophobic attributes of Angie 5 to inhibit TcdB, are our results of the in silico prediction of the TcdB-Angie 5 complex. The results revealed that the hydrophobic N-terminus of Angie 5 (LYS1-GLY9) seem to play an important role in stabilization of the TcdB-Angie 5 complex within the hydrophobic pocket formed at the interface between the GTD, CPD, and DRBD domains. Moreover, the electrostatic and hydrophobic interactions established by the peptide through hydrogen bonds and salt bridges are highly stabilizing interactions where ARG12 is involved. When modifying Angie 5 in its predicted binding pocket into the other tested Angie peptides, only Angie 3 exhibited a comparable binding energy, while the other Angie variants provided lower affinities within the pocket of Angie 5. This suggests that the presence of isoleucine on position 5 may play a key role in the stabilization of the interaction and contributes to a higher binding affinity. Therefore, Angie 5 bears amino acids at positions 2, 5 or 12 that are favorable for the stabilization of the TcdB-Angie 5 complex and thus the mediation of the inhibitory effect of Angie 5 towards TcdB.

So far, no research team besides Noschka and colleagues, has tested the Angie peptides for their antimicrobial capacities. However, murine angiogenin 1 and 4 (Ang1 and Ang4), as well as human angiogenin, have been shown to have microbicidal activity against systemic bacterial and fungal pathogens, suggesting them to function as mediators during innate immune response after infection [25]. Moreover, a further study has shown that Ang4 possesses antimicrobial properties against *Salmonella typhimurium* LT2 by disrupting the bacterial membrane integrity, while the ribonuclease activity was shown not to be responsible for the antimicrobial activity [48].

Since the Angie peptides were initially designed to possess antimicrobial properties, further optimization of Angie peptides can be performed based on the results presented in this study, specifically to inhibit bacterial toxins, such as TcdA and TcdB. However, the strongest antimicrobial effect was obtained for Angie 5, followed by Angie 3 and Angie 1, while the other Angie peptides showed no antimicrobial effect towards a non-toxin producing *C. difficile* strain. When performing transmission electron microscopy, we observed that Angie 5 disrupted the bacterial cell membranes, contributing to cytoplasmic leakage and thus, cell death. The disruption of bacterial membrane integrity displays a common mechanism to kill bacterial cells, as it has been reported earlier, for example for peptides derived from endogenous β−2 microglobulin and other peptides e.g., LL-37 and derivatives [24, 43]. Similar to these antimicrobial peptides that interact with or disrupt bacterial cell membranes, the Angie peptides as well have a positive net charge and thus are cationic. Cationic and hydrophobic peptides, such as the Angie peptides, can interact with the anionic bacterial plasma membrane of Gram-negative and -positive bacteria [24, 35, 58]. It is not surprising, when investigating the antimicrobial effect of the Angie peptides towards ESKAPE pathogens, further bacterial species were inhibited by the Angie peptides. Namely, an inhibition of *P. aeruginosa* and *A. baumannii* by Angie 1, 3, and 5 was observed, but not by Angie 6, 7, and the reference Angie. Consequently, targeting both, the bacterium, in specific *C. difficile* and its bacterial toxins, TcdA and TcdB might be beneficial in a clinical setting to inhibit not only the toxin but also the toxin-producing bacterium. Future mechanistic studies are planned to further elucidate the antimicrobial mode of inhibition of Angie 5 against toxin producing *C. difficile* strains and its pharmacological delivery. Noschka et al. have already shown that Angie 1 can be efficiently delivered into human macrophages via liposomes in prospects of the treatment of *Mycobacterium tuberculosis* [38]. Moreover, in the same study, Angie 1 was not toxic for zebrafish embryos and the serum stability of Angie 1 was assessed. The calculated half-life of Angie 1 using mass spectrometry using an Orbitrap Elite System was 3.058 min [38]. Within our study, the half-life of Angie 5 was 5.441 min and determined via MALDI-TOF. The usage of different methods does not allow a direct comparison, however for both Angie 1 and Angie 5 the half-life was short with a few minutes. As stability of peptides for in vivo application is a major concern, in the future, Angie 5 could be optimized by introducing chemical modifications, including D-amino acids or unnatural/non-coding amino acid substitutions, end-group capping, cyclization, and N/C-terminal modifications or substitutions e.g., to macromolecules, encapsulation, or further approaches [10]. As of now the terminal groups of the Angie peptides are NH_2_- and -COOH, from the N-terminal residue (K) and the C-terminal residue (I), respectively. Therefore, the residues are free and possess no modification.

In our performed experiments, Angie 5 showed a robust inhibition of TcdB in three cell lines including, HeLa cells, Vero cells, and the more physiologically relevant human colon carcinoma cell line CaCo-2. Moreover, Angie 5 showed inhibition of TcdA in Vero cells and protects Vero cells from the cytotoxic effects of the medically relevant combination of TcdA and TcdB. This makes Angie 5 an interesting candidate for further optimization regarding of drug development. To further assess the clinical relevance of Angie 5 as an antitoxin inhibitor, further studies could examine the protective effect of Angie 5 towards the hypervirulent strains BI/NAP1/027 of *C. difficile*, which express slightly modified versions of TcdA and TcdB and has emerged recently.

## Experimental procedures

### Compounds and reagents

The native toxins TcdA and TcdB from *C. difficile* VPI 10,463 were generously provided by Klaus Aktories (University of Freiburg, Germany) and purified as described earlier [29]. The peptide α-defensin-6 was purchased from PeptaNova (Sandhausen, Germany).

### Peptide design

The antimicrobial segment of angiogenin was predicted as previously shown [7] by AMPA, http://tcoffee.crg.cat/apps/ampa/do [49, 50], CAMP_R3_, http://www.camp.bicnirrh.res.in/prediction.php [54] using the Rational Design of Antimicrobial Peptides tool, and then evaluated by CAMP_R3_-Predict Antimicrobial Peptide tool, Antibp2, http://crdd.osdd.net/raghava/antibp2/ [32], ClassAMP, http://www.bicnirrh.res.in/classamp/predict.php [27], Peptide AMP Scanner, https://www.dveltri.com/ascan/v2/ascan.html [51], and iAMPpred, http://cabgrid.res.in:8080/amppred/server.php [37].

### Synthesis of Angie peptides

The Angie peptides Angie 1, 3, 5, 6, and 7 were obtained from PSL Heidelberg (PSL, Heidelberg, Germany) using F-moc chemistry [38].

The reference Angie was synthesized on site (CFP, Ulm, Germany) as previously described by Harms et al. [22]. Briefly, the peptides were synthesized via standard Fmoc solid-phase peptide synthesis using a Liberty Blue microwave synthesizer (CEM Corporation, Matthews, NC, USA) and then purified using reversed-phase high-performance liquid chromatography (Waters, Milford, MA, USA), employing an acetonitrile/water gradient under acidic conditions on a Phenomenex C18 Luna column (particle size 5 μm, pore size 100 Å). The pure peptide was lyophilized on a freeze-dryer (Labconco, Kansas City, MI, USA), and the molecular mass was verified by liquid chromatography–mass spectrometry (LC-MS; Waters, Milford, MA, USA). For the experiments the peptides were dissolved in water.

### Half-life determination of Angie 5 in human plasma

The half-life of Angie 5 in plasma was calculated according to a work by Freisem et al. [15], with minor modifications. A sample (0.5 ml) of human plasma was spiked with 20 µM Angie 5 and incubated at 37 °C. Aliquots (50 µl) were separated at 0, 15, 30, 60, and 120 min, respectively, and mixed with 250 µl 0.1% TFA in acetonitrile at − 20 °C. The mixture was centrifuged at 13,000 rpm for 30 s, and 150 µl of the supernatant was mixed with 150 µl 20% acetic acid in ice. The samples were analyzed with an Axima Confidence MALDI-TOF MS (Shimadzu) in linear mode using exactly the same measurement conditions for all samples spotted on a 384-well plate. Wells were coated with 0.5 µl of 10 mg/ml CHCA previously dissolved in TFA/water/acetonitrile/2-propanol (2.5/47.5/25/25, v/v), and the solvent was allowed to evaporate. Then, each sample (0.5 µl), previously mixed with matrix (0.5 µl), was applied onto the dry pre-coated well, and the solvent was allowed to evaporate. Laser shots were automatically done following a regular circular raster of a diameter of 2000 μm and spacing of 200 μm on each well; 100 profiles were acquired per sample, and 20 shots were accumulated per profile. An accelerating voltage of 20 kV was applied to the ion source. Measurements of each sample were done in triplicate. The measurement and MS data processing (peak area calculation) were controlled with MALDI-MS Application Shimadzu Biotech Launchpad 2.9.8.1 (Shimadzu). The half-life was calculated using GraphPad Prism version 10.3.1 for Windows, GraphPad Software, Boston, Massachusetts USA, www.graphpad.com. Data (signal area vs. time) was fitted to a one-phase decay curve.

### Bacterial culturing

All bacterial strains used for the susceptibility testing are listed in Supplementary Table 1. The *C. difficile* strain used is the strain VPI 11186 and PCR negative for *cdtB*, *tcdA*, and *tcdB* genes. All bacteria were cultivated on Tryptone Soya Agar with Sheep Blood (Thermo Scientific, Waltham, MA, USA) at 37 °C and 5% CO_2_. *C. difficile* was cultivated under anaerobic conditions, created by an GENbag anaer bag (bioMérieux, Marcy-l’Étoile, France). Liquid cultivation of *C. difficile* was performed in Brain-Heart infusion medium (Oxoid, Dardilly, France) supplemented with 0.5% yeast (Gibco) and 0.4 g/l L-cysteine (Fluka-Honeywell Research Chemicals, Morris Plains, NJ, USA) (BHI). After inoculation, the liquid culture was overlayed with sterile liquid Vaseline (VWR, Radnor, PA, USA) to achieve anaerobic conditions. For liquid cultivation of *P. aeruginosa*, *A. baumannii*, and *E. coli*, bacteria were inoculated in lysogeny broth (LB-Miller) and incubated at 37 °C with shaking at 160 rpm. *E. faecium*, *S. aureus*, and *K. pneumoniae* were grown in Todd-Hewitt Broth (Oxoid, Dardilly, France) supplemented with 5% yeast at 37 °C and 5% CO_2_.

### Radial diffusion assay

To investigate antimicrobial activity of the Angie peptides an overlay-assay was performed, as previously described [52]. In short, bacteria were inoculated in liquid agarose with a density of 2 × 10^7^ cells per plate. Wells were put into the solidified 1% agarose and filled with the different Angie peptides (100 µM). After 3 h of incubation at 37 °C an overlay with nutrient agar was performed. Plates were incubated at 37 °C and 5% CO_2_ and inhibition zones were measured after overnight incubation. For *C. difficile*,* s*ome modifications were made to account for the anaerobic conditions. In detail, 1 ml of a *C. difficile* overnight culture was directly added the agarose, mixed and a plate was poured. After drying for 5 min at 4 °C, wells were put in the agarose and filled with 10 µl of the Angie peptides 1, 3, and 5 in concentrations ranging from 100 µM to 1 mM or the various Angie peptides (5 mM and 100 µM for the reference Angie). The plate was incubated at 37 °C in a GENbag anaer bag for 3 h, then an overlay with 10 ml BHI-Agar was conducted. After overnight incubation, inhibition zones were measured. As a positive control LL-37 (Anaspec, Fremont, CA, USA) was used at a concentration of 1 mg/ml for *C. difficile* and 100 µg/ml for all other bacteria.

### Transmission Electron microscopy of C. difficile

To investigate effects of Angie 5 on *C. difficile*, transmission electron microscopy was performed, as previously described [20]. Shortly, *C. difficile* was grown for 3 h and cells were harvested by centrifugation (2 min, 8800 xg). The pellet was reconstituted in 10 mM phosphate solution and either Angie 5 or water was added. For the incubation period (37 °C, 1 h), an overlay with sterile liquid Vaseline was performed to ensure anaerobic conditions. The samples were subsequently fixed using 3.5% glutaraldehyde, 1% saccharose in phosphate buffer. Bacterial cells were postfixed in osmium tetroxide and dehydrated in a graded series of propanol. Finally, cells were stained with uranyl acetate, embedded in Epon and ultrathin sections were prepared using standard procedures. A Jeol 1400 Transmission Electron Microscope was used to analyze the samples and at least 25 pictures per sample were taken. The experiment was conducted once.

### Cell lines

All materials for the cultivation of cell lines were purchased from Gibco (Thermo Fisher Scientific, Waltham, MA, USA), unless indicated differently. The experimentally used cell lines included Vero cells (African green monkey kidney cells; DSMZ, Braunschweig, Germany), HeLa cells (human cervical carcinoma cells; DSMZ, Braunschweig, Germany), and CaCo-2 cells (human epithelial colorectal adenocarcinoma cells, ATCC HTB-37, Manassas, VA, USA) which were cultivated under humidified conditions at 37 °C with 5% CO_2_. The cells were trypsinized and reseeded every two to three days with a maximum of 25 times, while Vero cells and HeLa cells were cultivated in minimum essential medium (MEM), supplemented with 10% FCS, 1 mM sodium pyruvate, 0.1 mM non-essential amino acids and 100 U/ml penicillin and 100 g/ml streptomycin. CaCo-2 cells were cultivated in Dulbecco’s Modified Eagle Medium (DMEM), supplemented with 10% FCS, 1 mM sodium pyruvate, 0.1 mM non-essential amino acids and 100 U/ml penicillin and 100 g/ml streptomycin. For the performed intoxication experiments, cells were seeded in respective culture dishes one or two days before and treated in FCS-free media with toxins and the respective compounds.

### Cytopathic cell rounding assay

For the analysis of cell rounding due to intoxication with TcdA and or TcdB cell morphology assays were conducted. Therefore, the respective cell line was seeded in 96-well plates one or two days prior to treatment with toxins, Angie peptides or water (solvent control) in FCS-free medium. The treated cells were incubated using humidified conditions at 37 °C with 5% CO_2_. Afterwards, the cell morphology was monitored using light microscopy every hour for at least 6 h using a Leica DMi1 microscope connected to a Leica MC170 HD camera (both Leica Microsystems GmbH, Wetzlar, Germany). Rounded and non-rounded cells were counted using the online software Neuralab (https://neuralab.de).

### Glucosylation status of intracellular Rac1

For the analysis of the glucosylation status of intracellular Rac1, Vero cells were seeded one day before the treatment with toxin, Angie peptides or water (solvent control) in 24 well plates in FCS-free medium. After treatment, the cells were incubated with the approaches for 2 h or according to incubation times of the time series. At the end of the intoxication time, whole-cell lysates were harvested in 2.5x Laemmli (0.3 M Tris-HCl, 10% SDS, 37.5% glycerol, 0.4 mM bromophenol blue, 100 mM DTT) and samples were heat-denatured at 95 °C for 10 min. Afterwards, samples were subjected to SDS-PAGE and immunoblotting.

### Gel electrophoresis and Immunoblotting

After sample preparation SDS-PAGE and immunoblotting was performed. For separation of proteins gel electrophoresis was conducted, using 8 or 12.5% acrylamide gels, depending on the size of the protein of interest. Subsequently, semi-dry Western blotting was performed for the transfer of proteins from the gels onto nitrocellulose membranes, which was controlled by staining the membranes with Ponceau-S (AppliChem GmbH, Darmstadt, Germany). Next, the membranes were blocked at room temperature, using 5% skim milk powder in PBS-T (PBS containing 0.1% Tween 20) for at least 30 min. After blocking, washing steps were performed in PBS-T, followed by incubation with primary and secondary antibodies, again separated by washing steps with PBS-T. For detection of non-glucosylated Rac1 a respective primary antibody was used, primary mouse anti-Rac1 antibody (1:1000, #610651; clone 102; BD Biosciences, Heidelberg, Germany), while Hsp90 was detected as loading control, using a primary mouse anti-Hsp90 antibody (1:1000, Santa Cruz Biotechnology, Heidelberg, Germany). Total Rac1 was detected using the primary mouse anti-Rac1 antibody (Clone 23A8, Sigma-Aldrich Chemie GmbH, Germany). As secondary antibodies Horseradish peroxidase (HRP)-coupled goat anti-mouse IgG (H + L) secondary antibody (#31430; Thermo Fisher Scientific, Waltham, USA) and HRP-coupled mouse IgG kappa-binding protein (m-IgGκ BP-HRP; Santa Cruz Biotechnology, Dallas, USA; sc −516,102) were used respectively. After incubation with secondary antibodies, washing steps were performed and signals were detected using Pierce ECL Western blotting substrate (Thermo Fisher Scientific, Waltham, MA, USA), using the iBright 1500 system (Thermo Fisher Scientific). The signal quantification was performed using the ImageJ software v.1.52.a (NIH).

### In vitro glucosylation status of Rac1 from Whole-Cell lysates

For the analysis of the in vitro glucosylation status of Rac1, cell lysate was generated from CaCo-2 cells. Therefore, CaCo-2 cells were seeded in 10 cm culture dishes and grown for two to three days. Afterwards, cells were washed and frozen for cell lysis. Cell lysates were collected in glucosylation buffer (50 mM HEPES, 100 mM KCl, 2 mM MgCl_2_, 1 mM MnCl_2_, 100 mg/L BSA, pH 7.5) or in glucosylation buffer without BSA (50 mM HEPES, 100 mM KCl, 2 mM MgCl_2_, 1 mM MnCl_2_, pH 7.5), centrifuged at 10,000 x g for 1 min, the supernatant was transferred into a new tube, and the protein concentration was determined at the Nanodrop. The Angie peptides or water (solvent control) and 10 nM TcdB were mixed in glucosylation buffer with or without BSA and directly added to 40 µg CaCo-2 cell lysate. Subsequently, the samples were incubated for 2 h at 37 °C. After that, the samples were subjected to gel electrophoresis and immunoblotting. Rac1, total Rac1, and Hsp90 as a loading control were detected as described above and signals were quantified using the ImageJ software v.1.52.a (NIH).

### Actin-Staining and fluorescence microscopy

For analysis of the cytoskeletal modification upon toxin treatment staining and fluorescence microscopy experiments were performed where Vero cells were seeded and grown for one day in 18-well µ-slides (ibidi GmbH, Gräfelfing, Germany). Therefore, cells were treated with Angie peptides or water (solvent control) and intoxicated with TcdB in FCS-free medium for 2 h at 37 °C. After treatment, the cells were washed with PBS, fixed with 4% paraformaldehyde for 20 min, permeabilized using 0.4% (v/v) Triton X-100 in PBS for 5 min if required, and quenching was performed for 2 min in glycine (100 mM in PBS). This was followed by a blocking step for 1 h at 37 °C in PBS-T (PBS containing 0.1% Tween 20) containing 10% normal goat serum (Jackson ImmunoResearch, West Grove, PA, USA) and 1% BSA. For visualization of the cytoskeleton, F-actin was stained for 1 h at 37 °C, using the membrane-permeant SiR-actin (SiR-actin kit, Spirochrome, Stein am Rhein, Switzerland). Finally, cell nuclei were stained for 5 min using Hoechst 33,342 (1:10,000, Thermo Fisher Scientific, Waltham, MA, USA). After completing the staining procedure, the slides were examined via fluorescence microscopy using the BZ-X810 Keyence fluorescence microscope with a Plan Apochromat 40X objective and BZ-X filters (Keyence Deutschland GmbH, Neu-Isenburg, Germany) and BZ-X800Viewer v1.3.0.

### Cellular binding assay

For the analysis of TcdB binding to cells, Vero cells were seeded one day before treatment in 24 well plates. Then, cells were put on ice for 30 min and subsequently, Angie peptides or water (solvent control), and TcdB were added to cells and incubated for 1 h on ice in FCS-free medium. Cell lysates were collected in 2.5x Laemmli and samples were heat-denatured at 95 °C for 10 min. Afterwards, samples were subjected to gel electrophoresis and immunoblotting, while TcdB was detected, using an anti-TcdB antibody (1:1000, Anti-Clostridium difficile Toxin B antibody, Abcam, Cambridge, UK) and Hsp90 served as loading control. Signals were quantified using the ImageJ software v.1.52.a (NIH).

### In vitro precipitation assay

For the precipitation analysis, TcdB (50 ng equals 2 ng/µl) and inhibitors, α-defensin-6 (6 µM) and Angie peptides or water (solvent control) were incubated for 30 min in a total volume of 25 µl PBS. Next, the samples were centrifuged for 20 min, 14,000 rpm at 4 °C for separation of supernatant and pellet fraction. Therefore, the supernatant was transferred into a new tube and the pellet was resuspended in PBS. The samples were subjected to gel electrophoresis and immunoblotting. TcdB was detected using an anti-TcdB antibody (1:1000, Anti-Clostridium difficile Toxin B antibody, Abcam, Cambridge, UK).

### In Silico prediction of the complex TcdB-Angie 5

The structural prediction of the TcdB-Angie 5 complex was carried out using three web-based tools: AlphaFold3 (https://alphafoldserver.com/) [1], HPEPDOCK (http://huanglab.phys.hust.edu.cn/hpepdock/) [57], and PEP-SiteFinder (https://bioserv.rpbs.univ-paris-diderot.fr/services/PEP-SiteFinder/) [44]. In all cases, the TcdB protein was designated as the receptor, while the Angie 5 peptide served as the ligand. The amino acid sequence and three-dimensional (3D) structure of TcdB were retrieved from the Protein Data Bank [8] under the accession code 6oq5 [12].

For AlphaFold3, the receptor and peptide sequences were provided as input, whereas for HPEPDOCK and PEP-SiteFinder the 3D structure of TcdB and the peptide sequence were used. All docking simulations were performed in a blind manner using the default parameters of each server. Since AlphaFold3 generates only five docking models per run, the docking procedure was repeated three times with this tool. The resulting models from each server were clustered based on a root-mean-square deviation (RMSD) threshold of 20 Å, yielding the ten most representative clusters per server. The best structure from each cluster was selected based on the scoring function of the respective server.

To facilitate comparison and identify the most stable complex, the binding energy of the selected structures was re-evaluated using the Prodigy server (https://rascar.science.uu.nl/prodigy/) [56]. The TcdB-Angie 5 complex with the lowest predicted binding energy was considered the most probable conformation. Interaction analysis of the selected complex and the identification of key binding residues (hot spots) in the peptide were performed using PPCheck (https://caps.ncbs.res.in/ppcheck/) [47].

To validate these findings, in silico mutagenesis was conducted by manually modifying the Angie 5 peptide within the complex to generate the other Angie peptide variants (Table 1). The binding energy of these TcdB-peptide complexes was subsequently calculated using Prodigy and compared with experimental data from inhibitory assays.

### In vitro autoprocessing assay of TcdB

To investigate the effects of the Angie peptides on the intrinsic cysteine protease activity of TcdB an in vitro autoprocessing assay of TcdB was conducted. TcdB with or without the different Angie peptides was incubated for 1 h at 37 °C in a 20 mM Tris-HCl buffer containing 150 mM NaCl at pH 7.4. The autoprocessing activity was induced by the addition of 1 mM inositol hexakisphosphate (Santa Cruz Biotechnology). To inhibit the autoprocessing of TcdB, a positive control containing 1 mM N-ethylmaleimide (NEM) (Sigma Aldrich by Merck) was added. The reaction was stopped by the addition of Laemmli buffer. The samples were incubated for 10 min at 95 °C and subjected to SDS-PAGE and immunoblotting, while TcdB was detected, using an anti-TcdB antibody (1:1000, Anti-Clostridium difficile Toxin B antibody, Abcam, Cambridge, UK).

### Reproducibility of experiments and statistics

All performed experiments were conducted independently from each other at least three times. The number of replicates (n) for experiments or tested conditions is given in the figure legends, while representative results are shown in the figures. If not stated otherwise in the figure legends, the statistical analysis performed was a one-way ANOVA in combination with Dunnett’s multiple comparison test using GraphPad Prism Version 9 (GraphPad Software Inc., San Diego, CA, USA). The obtained p values are depicted as follows: ns = not significant *p* > 0.05, * *p* < 0.05, ** *p* < 0.01, *** *p* < 0.001, **** *p* < 0.0001.

## Electronic supplementary material

Below is the link to the electronic supplementary material.

Supplementary Material 1

## Data Availability

Any data and materials reported in this paper are available from the corresponding author upon reasonable request.

## Abbreviations

AB: *Acinetobacter baumannii*
CDADs: *C. difficile*-associated diseases
CDI: *C. difficile *infections
CGTs: Clostridial glucosylating toxins
C: *Clostridioides difficile*
CPD: Cysteine protease domain
E: *Enterococcus faecium*
E: *Escherichia coli*
GTD: Glucosyltransferase domain
IC_50_: Half-maximal inhibitory concentration
IB: Immunoblotting
InsP6: Inositol hexakisphosphate
K: *Klebsiella pneumoniae*
LM: Light microscope
MALDI-TOF: Matrix-assisted laser desorption ionization-time of flight
NEM: N-ethylmaleimide
P: *Pseudomonas aeruginosa*
RMSD: Root-mean-square deviation
S: *Staphylococcus aureus*
TcdA: Toxin A
TcdB: Toxin B
V-ATPases: Vesicular adenosine triphosphatases
