# Supplementary material for: The antimicrobial peptide Angie 5 inhibits TcdA and TcdB from Clostridioides difficile
Source: Cell Mol Life Sci. 2025 Jun 30;82(1):265. doi: 10.1007/s00018-025-05799-2 (PMC12209146; doi:10.1007/s00018-025-05799-2)
Supplement: Supplementary file 1 — Supplementary Material 1 [file 18_2025_5799_MOESM1_ESM.docx]

**Supplementary figures - The antimicrobial peptide Angie 5 inhibits TcdA and TcdB from *Clostridioides difficile***

**Supplementary Table 1. Information on the bacterial strains used for the radial diffusion assays.** (American Type Culture Collection is abbreviated as ATCC, Extended spectrum beta lactamase is shorted to ESBL, DSM stands for Deutsche Sammlung von Mikroorganismen, MRSA indicates methicillin resistant *Staphylococcus aures*, Vancomycin resistant enterococci are abbreviated as VRE)

| **Bacterial strain** | **Description** | **Source** |
| --- | --- | --- |
| *Pseudomonas aeruginosa*, BSU 856 | ATCC 27853 | ATCC |
| *Escherichia coli*, BSU 1286 | ESBL | Clinical Isolate, Ulm University Medical Center |
| *Acinetobacter baumannii*, BSU 1514 | ATCC 19606 | ATCC |
| *Klebsiella pneumoniae*, BSU 2231 | DSM 30104 | DSM |
| *Stapylococcus aureus*, BSU 1348 | MRSA, ATCC 43300 | ATCC |
| *Enterococcus faecium,* BSU 1516 | VRE, DSM 17050 | DSM |
| *Clostridioides difficile*, BSU 1671 | Toxin negative, ATCC 700057 | ATCC |

**Supplementary Figure 1. Antimicrobial activity of Angie peptides against *C. difficile*.** **(a)** For the radial diffusion assay an agar plate was prepared with a *C. difficile* overnight culture. Wells were put in the agarose and filled with 10 mg/ml Angie 1, 3, 5, 6, 7, and 3 mg/ml reference Angie. The plate was incubated at 37 °C for 3 hours and an overlay with BHI-Agar was conducted. After overnight incubation, inhibition zones were measured. As a positive control 1 mg/ml LL-37 was used. The inhibition zone is given in cm, mean +/- SEM (n = 4 - 5 values from five independent experiments). **(b)** Representative image for the radial diffusion assay.

**Supplementary Figure 2. Antimicrobial activity of Angie peptides against *P. aeruginosa* and *A. baumannii*.** **(a)** For the radial diffusion assay an agar plate was prepared with a *P. aeruginosa* and *A. baumannii* overnight culture. Wells were put in the agarose and filled with the different Angie peptides (100 µM). The plate was incubated at 37 °C for 3 hours and an overlay with nutrient agar was performed. After overnight incubation, inhibition zones were measured. As a positive control 100 µg/ml LL-37 was used. The inhibition zone is given in cm, mean +/- SEM (n = 5 values from five independent experiments). **(b, c)** Representative image for the radial diffusion assay for *P. aeruginosa* (b) and *A. baumannii* (c).

******

**Supplementary Figure 3. Effect of Angie peptides on TcdB mediated cell rounding of HeLa, Vero, and CaCo-2 cells. (a-c)** TcdB (10 pM/ 200 pM) and the different Angies (100 µM) or the respective amount of its solvent (H_2_O) were added together in FCS-free medium to HeLa (10 pM TcdB) (a), Vero (10 pM TcdB) (b), and CaCo-2 (200 pM TcdB) (c) cells. The cells were incubated for 7 h at 37 °C, and pictures were taken every hour. Representative pictures after 7 h are shown (n = 9 values from three independent experiments, each performed with triplicates (three independent wells)).

******

**Supplementary Figure 4. Effect of the reference Angie peptide on TcdB mediated cell rounding of Vero cells. (a)** Procedure of the cell morphology assay in a schematic representation. TcdB and the reference Angie was added simultaneously to cells and incubated for 7 h at 37 °C. Pictures were taken every hour using the light microscope (LM). **(b-d)** TcdB (10 pM) and the reference Angie (100 µM) or the respective amount of its solvent (H_2_O) were added together in FCS-free medium to Vero cells. The cells were incubated for 7 h at 37 °C, and pictures were taken every hour. Rounded cells are given as percent of the total cell count, mean +/- SEM (n = 9 values from three independent experiments, each performed with triplicates (three independent wells)), for the time course of the whole experiment (b) or for the endpoint after 7 h (c). Representative images are shown (d). Significance was tested using one-way ANOVA followed by Dunnett’s multiple comparison test and refers to TcdB treated controls (TcdB) (* p < 0.1, ** p < 0.01, *** p < 0.001, **** p < 0.0001, ns not significant).

******

**Supplementary Figure 5. Effect of preincubation of Angie 5 and TcdB on TcdB mediated cell rounding of Vero cells. (a)** Procedure of the cell morphology assay in a schematic representation. TcdB and Angie 5 were preincubated for 15 minutes at room temperature before addition to cells and incubated for 7 h at 37 °C. Pictures were taken every hour using the light microscope (LM). **(b-e)** TcdB (10 pM (b-e)) and the different concentrations of Angie 5 and the reference Angie (100 µM) or the respective amount of its solvent (H_2_O) were added together in FCS-free medium and incubated for 15 min at room temperature before addition to Vero cells (b-e). The cells were incubated for 7 h at 37 °C, and pictures were taken every hour. Rounded cells are given as percent of the total cell count, mean +/- SEM (n = 9 - 18 values from six independent experiments, each performed with triplicates (three independent wells)), for the time course of the whole experiment (b), after 3 hours (d-e) for the endpoint after 7 h (c). **(e)** The IC_50_ values for Angie 5 were calculated from the percentage of rounded cells of the total cell count after 3 hours of incubation. A nonlinear regression model with variable slope (GraphPad Prism, log(inhibitor) versus response (variable slope, four parameters)) was fitted to values, and IC_50_ values were given based on the fit. Significance was tested using one-way ANOVA followed by Dunnett’s multiple comparison test and refers to TcdB treated controls (TcdB) (* p < 0.1, ** p < 0.01, *** p < 0.001, **** p < 0.0001, ns not significant).

******

**Supplementary Figure 6. Effect of preincubation of Angie 5 and TcdB versus no preincubation on TcdB mediated cell rounding of Vero cells.** TcdB (10 pM) and the different concentrations of Angie 5 and the reference Angie (100 µM) or the respective amount of its solvent (H_2_O) were added together in FCS-free medium to Vero cells (left side) or preincubated for 15 min at room temperature before addition to Vero cells (right side). The cells were incubated for 7 h at 37 °C, and pictures were taken every hour. Rounded cells are given as x-fold of the total cell count and of control (TcdB), mean +/- SEM (n = 9 - 18 values from six independent experiments, each performed with triplicates (three independent wells)) after 3 hours. Data summarized and normalized to TcdB control from Figure 4 and Supplementary Figure 3. Significance was tested using one-way ANOVA followed by Šídák's multiple comparisons test (* p < 0.1, ** p < 0.01, *** p < 0.001, **** p < 0.0001, ns not significant).

******

**Supplementary Figure 7. Effect of Angie peptides on TcdA or TcdA and TcdB mediated cell rounding of Vero cells. (a-b)** TcdA (180 pM) (a) or TcdA (180 pM) with TcdB (10 pM) (b) and the different Angies (100 µM) or the respective amount of its solvent (H_2_O) were added together in FCS-free medium to Vero cells. The cells were incubated for 7 h at 37 °C, and pictures were taken every hour. Representative pictures after 7 h are shown (n = 9 values from three independent experiments, each performed with triplicates (three independent wells)).

**Supplementary Figure 8. Effect of Angie peptides on enzyme activity of TcdB *in vitro* using CaCo-2 cell lysate. (a)** Procedure of the enzyme activity assay analyzing the glucosylation status of Rac1 *in vitro* in a schematic representation. The different Angie peptides or the respective amount of solvent (H_2_O) and TcdB were added directly to CaCo-2 cell lysate. Then the samples were incubated for 2 h at 37 °C using glucosylation buffer without BSA and subsequently analyzed via SDS-PAGE and immunoblotting. **(b-c)** The different Angie peptides (100 µM) or the respective amount of solvent (H_2_O) and TcdB (10 nM) were added directly to CaCo-2 cell lysate (40 µg). After that samples were prepared and analyzed via SDS-PAGE and immunoblotting, while signals for non-glucosylated Rac1, total Rac1, and Hsp90 as loading control were detected and quantified. Values are given as mean +/- SEM (n = 5 - 11 values from four independent experiments) (b). Blot pictures for representative experiments are shown (c). Significance was tested using one-way ANOVA followed by Dunnett’s multiple comparison test and refers to TcdB treated controls (TcdB) (* p < 0.1, ** p < 0.01, *** p < 0.001, **** p < 0.0001, ns not significant).

**Supplementary Figure 9. Effect of the Angie peptides on TcdB mediated cell rounding of Vero cells in serum containing conditions. (a)** Procedure of the cell morphology assay in a schematic representation. TcdB and the Angie peptides were added simultaneously to cells in serum containing conditions and incubated for 7 h at 37 °C. Pictures were taken every hour using the light microscope (LM). **(b-d)** TcdB (10 pM) and the Angie peptides (100 µM) or the respective amount of its solvent (H_2_O) were added together in FCS-containing medium to Vero cells. The cells were incubated for 7 h at 37 °C, and pictures were taken every hour. Rounded cells are given as percent of the total cell count, mean +/- SEM (n = 9 values from three independent experiments, each performed with triplicates (three independent wells)), for the time course of the whole experiment (b) or for the endpoint after 7 h (c). Representative images are shown (d). Significance was tested using one-way ANOVA followed by Dunnett’s multiple comparison test and refers to TcdB treated controls (TcdB) (* p < 0.1, ** p < 0.01, *** p < 0.001, **** p < 0.0001, ns not significant).

**Supplementary Figure 10. Normalized binding energy for the best representative structures selected from each cluster of each docking software.**

**Supplementary Figure 11. Effect of the Angie peptides on the autoproteolytic activity of TcdB.** TcdB (50 ng) was incubated with or without the different Angie peptides and InsP6 in buffer for 1 h at 37°C. As further negative control TcdB was left untreated or as positive control for inhibition of the autoproteolytic activity of TcdB incubated with InsP6 and NEM. The samples were subjected to SDS-PAGE and immunoblotting, while a TcdB antibody was used also recognizing the GTD domain.

**Supplementary Table 2.** **Comparison between the theoretical binding energy of each TcdB-peptide complex and the experimentally determined potency.**

| **Peptide** | **Theoretical**  **Binding Energy [kcal/mol]** | **Experimental potency** |
| --- | --- | --- |
| Angiogenin 64-80  (reference Angie) | -16.4 | - |
| Angie 1 | -16.4 | + |
| Angie 3 | -18.8 | + |
| Angie 5 | -18.9 | ++ |
| Angie 6 | -16.3 | - |
| Angie 7 | -16.7 | - |

**Supplementary Figure 12. Comparison between the theoretical binding energy of each TcdB-peptide complex.**
